# An analysis about heterogeneity among cancers based on the DNA methylation patterns

**DOI:** 10.1186/s12885-019-6455-x

**Published:** 2019-12-30

**Authors:** Yang Liu, Yue Gu, Mu Su, Hui Liu, Shumei Zhang, Yan Zhang

**Affiliations:** 1 School of Life Science and Technology, Computational Biology Research Center, Harbin Institute of Technology, Harbin, 150001 China; 2 College of Bioinformatics Science and Technology, Harbin Medical University, Harbin, 150081 China; 3 College of Information and Computer Engineering, Northeast Forestry University, Harbin, 150040 China

**Keywords:** DNA methylation, Cancer, Epigenetic heterogeneity, Survival analysis

## Abstract

**Background:**

It is generally believed that DNA methylation, as one of the most important epigenetic modifications, participates in the regulation of gene expression and plays an important role in the development of cancer, and there exits epigenetic heterogeneity among cancers. Therefore, this study tried to screen for reliable prognostic markers for different cancers, providing further explanation for the heterogeneity of cancers, and more targets for clinical transformation studies of cancer from epigenetic perspective.

**Methods:**

This article discusses the epigenetic heterogeneity of cancer in detail. Firstly, DNA methylation data of seven cancer types were obtained from Illumina Infinium HumanMethylation 450 K platform of TCGA database. Then, differential methylation analysis was performed in the promotor region. Secondly, pivotal gene markers were obtained by constructing the DNA methylation correlation network and the gene interaction network in the KEGG pathway, and 317 marker genes obtained from two networks were integrated as candidate markers for the prognosis model. Finally, we used the univariate and multivariate COX regression models to select specific independent prognostic markers for each cancer, and studied the risk factor of these genes by doing survival analysis.

**Results:**

First, the cancer type-specific gene markers were obtained by differential methylation analysis and they were found to be involved in different biological functions by enrichment analysis. Moreover, specific and common diagnostic markers for each type of cancer was sorted out and Kaplan-Meier survival analysis showed that there was significant difference in survival between the two risk groups.

**Conclusions:**

This study screened out reliable prognostic markers for different cancers, providing a further explanation for the heterogeneity of cancer at the DNA methylation level and more targets for clinical conversion studies of cancer.

## Background

Recently, cancers are found to have become serious threats to human health. Through epidemiological study, experiments and clinical observations, researchers found that environment and behavior have significant effects on the occurrence of human malignant tumors. All kinds of environmental and hereditary carcinogenic factor can work in a synergistic or orderly manner in the induction of non-lethal DNA damage in cells, which leads to the activation of oncogenes and/or the inactivation of tumor suppressing genes. Moreover, substantial omics heterogeneity has been revealed for histologically homogeneous tumors in terms of genomics [1, 2], epigenomics [3], transcriptomics [4–6] and proteomics [7]. Actually, epigenetic modification plays an important role in the development of cancers.

Previous study has proved that epigenetic modification stands for the intersections of genes and environment [8–10]. Epigenetic modification can regulate the expression of genes without altering basic DNA sequence [8]. Despite increasing evidence which shows that epigenetic modifications are sensitive to environmental exposure (such as nutritional factors), the influence on epigenetic markers cast by genetic mutation has been spotted [11]. One of the most common epigenetic modifications is DNA methylation. It occurs when methyl is added to specific DNA base pairs, primarily in the background of cytosine dinucleotide (CpG). DNA methylation has been well explored and demonstrated to play essential roles in cellular processes such as regulation of gene expression [12]. According to the place where methylation takes place (such as genome and CpG islands) [13] and the level of DNA methylation, two classes are created, hypomethylation and hypermethylation. There are several most common used ways to analyze the patterns of DNA methylation: global, epigenetic genome range and candidate gene DNA methylation analysis.

Cancer is a type of disease with great genetic and epigenetic heterogeneity. So far, there have been lots of studies that confirm the feasibility of analyzing the epigenetic heterogeneity of cancers using DNA methylation patterns. For instance, it has been proved that DNA methylation heterogeneity is related to Prostatic Carcinoma [14], Low-stage Glioma [15], Esophageal Squamous Cell Carcinoma [16], and the clone of Hepatocellular Carcinoma [17]. In addition, new indicators of DNA methylation heterogeneity, such as epiallele load, Inconsistent Methylated Read Ratio and DNA Methylation Inference Regulatory Activity, are related to the clinical variables of Acute Myeloid Leukemia [18], Chronic Lymphoblastic Leukemia [19] and Sarcoma [20]. However, these researches are all based on the heterogeneity analysis of a single type of cancer, it is also required for a pan-cancer heterogeneity analysis from the global perspective.

This study analyzes the heterogeneity of seven TCGA cancers based on DNA methylation level. We first define specific differentially methylated genes in these cancers. Then, we build methylation-correlation network and KEGG pathway network to sort out pivotal genes and find out cancer-specific methylation markers and prognostic markers. This research can provide clinicians and researchers with more therapeutic and experimental targets, and deeper understandings on cancer heterogeneity.

## Methods

### Acquisition and preprocessing of DNA methylation data

DNA methylation data of seven cancer types, including 337 COAD (colon adenocarcinoma) samples, 492 LUAD (lung adenocarcinoma) samples, 415 LUSC (lung squamous cell carcinoma) samples, 195 PAAD (pancreatic cancer) samples, 202 ESCA (esophageal cancer) samples, 888 BRCA (Breast invasive carcinoma) samples, 478 UCEC (Uterine Corpus Endometrial Carcinoma) samples, were downloaded from the TCGA (The Cancer Genome Atlas) database, Illumina Infinium HumanMethylation450 BeadChip platform. Specific sample information for each cancer type was shown in Table 1.

**Table 1:** The sample size for each cancer type

Cancer Type	Normal sample size	Cancer sample size	Stage (I / II / III / IV)
BRCA	98	790	138 / 452 / 211 / 22
COAD	38	299	56 / 128 / 98 / 53
ESCA	16	186	42 / 102 / 79 / 32
LUAD	32	460	255 / 117 / 78 / 25
LUSC	43	372	177 / 140 / 61 / 9
PAAD	10	185	23 / 153 / 7 / 8
UCEC	46	432	264 / 43 / 101 / 24

Some pre-processing is conducted on the DNA methylation data. We have removed samples with multiple missing values and recalculated missing values of remaining samples with the function impute. Knn (), R package. We also removed the unstable loci in genome, including CpG loci on sex chromosome, single nucleotide polymorphisms, and CpG loci corresponding to multiple genes. Since the methylation of CpG loci on the promotor region has a strong regulatory effect on gene expression, we only select the CpG loci in the promoter region of genes for further analysis. Here, the promoter region of the gene is defined as the upstream 2 kb region of the transcription initiation site to the downstream 0.5 kb region.

The chip HM450K checks the methylation level of over 480,000 CpG loci in the whole genome. Therefore, chances are that multiple CpG loci are tested in a single gene. Sometimes, differences are huge among those CpG loci which correspond to the same gene, so it’s not reasonable for all the genes we study, to use the average methylation level of those CpG loci to represent the methylation level of the gene. Zhang et al. propose that most of the CpG loci are hypermethylated or hypomethylated (β > 0.5 or β < 0.5) [21], hence in a single sample, we believe that the CpG loci on a gene (gene A) are of the same pattern if all of their β values are greater than or equal to (or less than) 0.5. It is reasonable for us to use the average methylation level of all the CpG loci on gene A to represent the methylation level of this gene if the ratio of samples of the same pattern reach a specific threshold. For genes don’t meet the condition, we remove them from the subsequent analysis. Finally, we use the average methylation level of all the CpG loci on a gene to represent the methylation level of it.

### Differentially methylated genes identification per cancer

DNA methylation is the most extensively documented epigenetic modification that can influence cell fate and gene expression [22], which finally leads to the inhibition of gene expression through formation of heterochromatin in the gene regulatory region [23]. In this study, identification of differentially methylated genes in cancer samples and adjacent control samples for all seven types of cancer are our first task.

We use user-defined R script, the bilateral t-test, to recognize the differentially methylated genes among sample pairs. Benjamini-Hochberg method is used in multiple tests to adjust the *P* value. The gene whose adjusted P value is less than 0.05 and the difference of the average of β is more than 10% is considered distinctly differentially methylated gene among sample pairs.

### Biological functions and pathways enrichment analysis of differentially methylated genes

In this study, using DAVID [24, 25], we conduct a GO (Gene Ontology) biological functions enrichment analysis and a KEGG (Kyoto Encyclopedia of Genes and Genomes) pathways enrichment analysis towards the list of differentially methylated genes from the seven cancer types (hypermethylated and differentially hypomethylated genes are also included), with p controlled within 0.05, which could find out the biological characteristics and senses related.

### Construction of correlation network of differentially methylated genes

In this study, Pearson correlation coefficient is used to measure the correlation of DNA methylation level of differentially methylated genes of each cancer type quantitatively. The formula is as follows:

$$ \mathrm{r}=\frac{1}{n-1}\sum \limits_{i=1}^n\left(\frac{X_i-\overline{X}}{\delta_X}\right)\left(\frac{Y_i-\overline{Y}}{\delta_Y}\right) $$

T test is used to perform a hypothesis test towards correlation coefficient. In addition to that, we also use permutation test to examine the correlation between DNA methylation levels in each pair of genes. Script of python and R are used to complete the process, and we use the function cor.test() in R for calculation and test of correlation coefficient. We build a methylation correlation net. This net is built, analyzed and visualized using Cytoscape 2.8.2 [17] (http://www.cytoscape.org/). The statistical and functional significance of the network, is proposed to be measured using various statistical parameters, namely in the proposed case, degree (the number of edges per node) and average clustering co-efficient C(k), the ratio of the number of edges E of the node having a k degree with neighbors to the total possible number of such edges.

In the DNA methylation correlation network, different nodes are of different importance, for those whose degrees are large, they often are pivots of the network with lots of genes related to them. If they go abnormal, vertexes adjacent to them will be affected, leading to dysfunction of the pathway and causing cancer. We assume that those key nodes may be associated with the prognosis of cancer patients, thus we pick the top 20% nodes in the network as candidate genes for further analysis. It is also necessary to analyze the interaction information in the pathways of these differentially methylated genes from a functional perspective. DAVID online bioinformatics tools are used in the enrichment analysis of the pathways and functions involved those genes. The result is visualized using EnrichmentMap function in Cytoscape.

### Construction of KEGG pathway network of differentially methylated genes

In this study, XML format files of pathways enriched by differentially methylated genes in each cancer type are obtained from KEGG database. User-defined Perl script is used, <relation></relation> block is used to find the molecular interaction pairs within each pathway, <entry></entry> block is used to obtain information about the specific genes or compounds of each pair. Among all those interaction pairs, the interactions of real proteins are our only concern, therefore only ‘PPreal’ type of interaction pairs in relation remain undeleted. Then, the resulting interaction id is then converted into a gene symbol to facilitate visualization and analysis. The network of KEGG pathway is also built by Cytoscape 2.8.2 [26] (http://www.cytoscape.org/) to analyze and visualize the network.

We also pick the top 20% nodes whose degrees are the biggest as candidate genes for further analysis and have a discussion on the functions of those genes. DAVID online bioinformatics tools are used to conduct an enrichment analysis on the pathways and functions in which those genes are involve, the results are visualized using EnrichmentMap [27] in Cytoscape.

### The construction of prognostic model and survival analysis

In order to be accurate, all cancer patients in each cancer type were divided into two data sets on average in this study, a training set and a test set. The training set is used for establishment of models and screening of prognostic markers while the test set is for follow-up validation of screened prognostic markers. The division of two sets should meet the following criteria: (1) All samples are divided into training set and test set randomly. (2) There were no significant differences in age distribution, staging, follow-up time and mortality between the two sets (Use Fisher’s exact test or t test). That is to say, patients of all types were randomly assigned to the training and test sets, including patients with missing clinical information. Then, we use the samples of each cancer in the training set and the differentially methylated candidate prognostic markers in each type of cancer obtained from correlation network and the KEGG pathway network to construct a model to screen for specific prognostic markers in cancers.

In the first step, we find out DNA methylation spectrum of candidate markers for each cancer type, as well as clinical phenotype information and follow-up information of the samples and establish a univariate COX proportional risk regression model, so as to assess the association between patient survival and DNA methylation levels. Additionally, we also construct univariate COX proportional risk regression models to determine the clinical factors that significantly affect patient survival. In the next step, significant genes in each cancer type and the clinical factors that significantly affect survival in this cancer type are introduced into the multivariate COX proportional risk regression model to find independent prognostic factors (genes). For each gene i, the formulas of univariate and multivariate COX proportional risk regression models are defined as follows:

$$ {\displaystyle \begin{array}{c}\mathrm{h}{\left(t,x\right)}_i=h0(t)\exp \left({\beta}_{methy} meth{y}_i\right)\\ {}\mathrm{h}{\left(t,x\right)}_i=h0(t)\exp \left({\beta}_{methy} meth{y}_i+\sum {\beta}_{clinical} clinical\right)\end{array}} $$

In the formula, *methy*_*i*_ is the DNA methylation level vector of Gene i in all Samples, clinical represents clinical attribute information, *β*_*methy*_, *β*_*clinical*_ are the coefficients of the regression model. The positive regression coefficient indicates that the increase of methylation level is related to the increase of death risk (risk gene), while the negative regression coefficient indicates that the increase of methylation level is related to the decrease of death risk (protective gene). Univariate and multivariate COX proportional risk regression models are constructed using function coxph() in survival R package.

After univariate and multivariate COX proportional risk regression analysis, independent prognostic markers that are still significant are used to calculate risk scores in the training set. Risk score is a linear combination of DNA methylation level and regression coefficient of these markers, representing different risk levels of patients. The formula is as follows:

$$ \mathrm{Risk}\kern0.17em \mathrm{Score}={\sum}_{i=1}^{\mathrm{n}}{\beta}_i{X}_i $$

In the formula, *β*_*i*_ is the COX regression coefficient of Gene i in the training set, *X*_*i*_ is the methylation level of Gene i, n is the number of genes that have a significant impact on survival. Next, taking the median risk score as the threshold, the patients in the training set are divided into high-risk group and low-risk group. The survival difference between the two groups is analyzed, the overall survival status of patients is estimated by Kaplan-Meier method and the statistical significance of the difference is determined by log-rank test. Functions survfit() and survdiff() in survival R package are used in the process.

Then, the regression coefficients and the threshold of risk score from the training set are directly applied to the test set, and the patients in the test set are also divided into high-risk group and low-risk group. The prognostic differences between the two risk groups were assessed using the same method as in the training set.

## Results

### Heterogeneity of differentially methylated genes per cancer

**Fig. 1:**
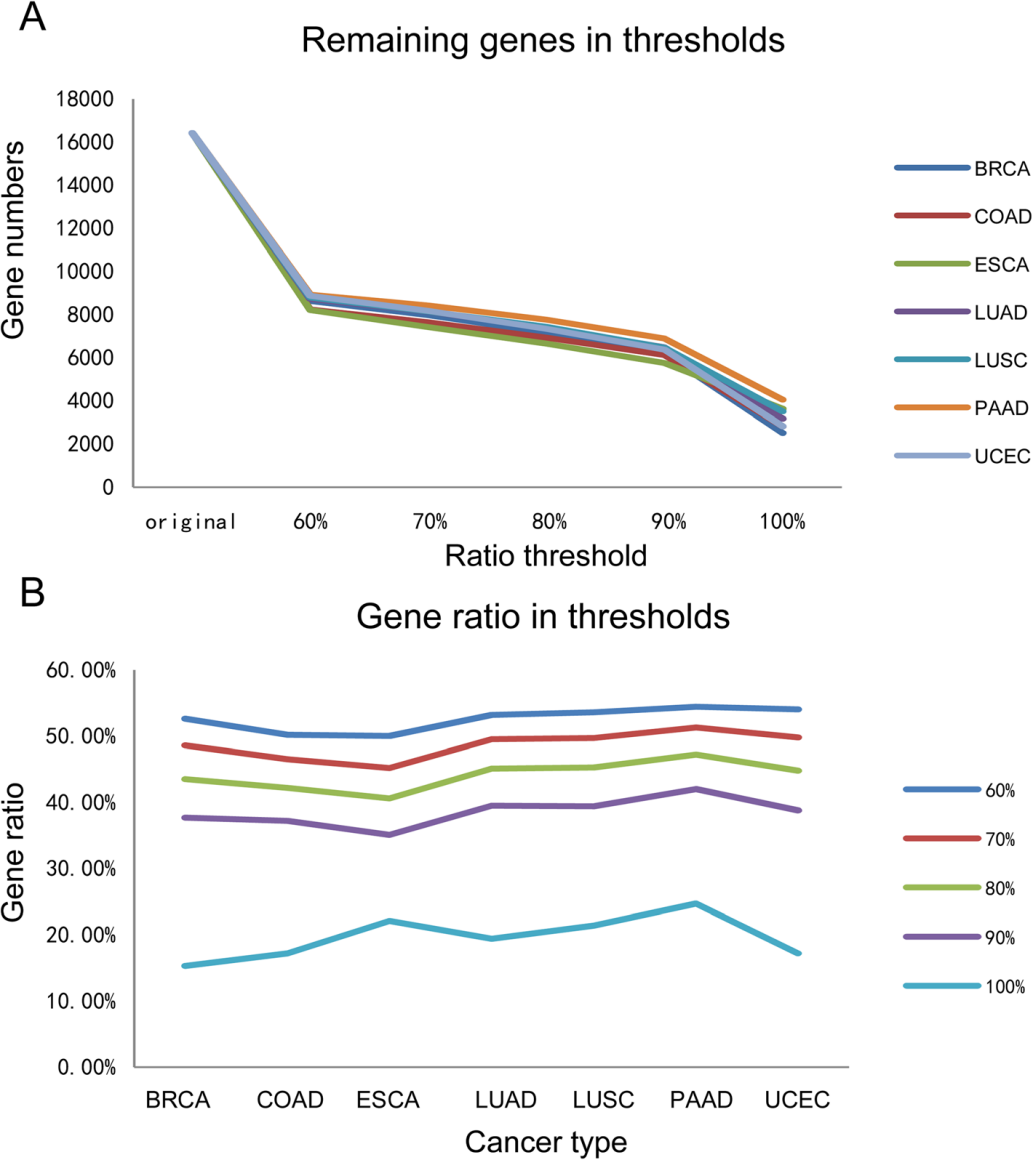
Comparison of the number of genes and the proportion of remaining genes obtained at different ratio thresholds. **a**. Comparison of the number of genes obtained at different ratio thresholds. **b**. Comparison of the proportion of remaining genes when different ratio thresholds are used

In this study, we have compared the number of the genes obtained and the proportion of the rest of the genes at different ratio threshold (Fig. 1). We hope that we could find a ratio threshold which retains as many genes as possible meanwhile improves the accuracy of calculation of gene methylation level. Eventually, we select 70% as the ratio threshold, which guarantees that about 50% of the original genes remain. At last, we use the average methylation of the CpG loci as the methylation level of the gene in further analysis.

**Fig. 2:**
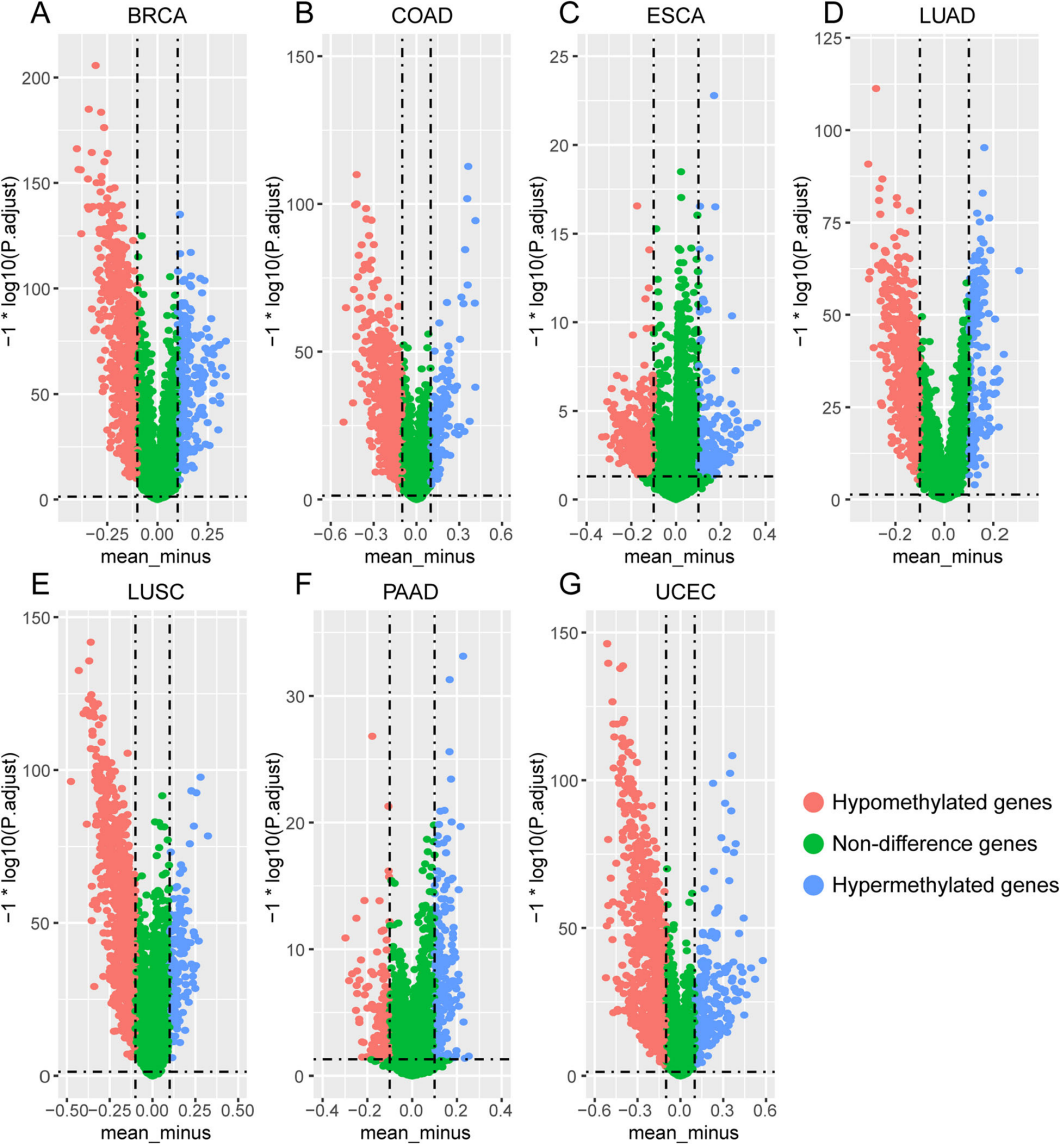
Volcano plot of differentially methylated genes in seven cancers. **a** Volcano plot of differentially methylated genes in BRCA. **b** Volcano plot of differentially methylated genes in COAD. **c** Volcano plot of differentially methylated genes in ESCA. **d** Volcano plot of differentially methylated genes in LUAD. **e** Volcano plot of differentially methylated genes in LUSC. **f** Volcano plot of differentially methylated genes in PAAD. **g** Volcano plot of differentially methylated genes in UCEC

Through the process mentioned above, we identified 2214 differentially methylated genes in the total seven cancer types. The numbers of hypomethylated and hypermethylated genes are shown in Table 2. The differentially methylated genes are shown in the volcano plot (Fig. 2), which is drawn using ggplot2 R package.

**Table 2:** The numbers of differential methylated genes in 7 cancer types

Cancer types	Number of genes	Number of differentially hypermethylated genes	Number of differentially hypomethylated genes
BRCA	7981	223	605
COAD	7643	159	547
ESCA	7423	177	396
LUAD	8133	181	542
LUSC	8153	170	901
PAAD	8430	183	159
UCEC	8170	233	813

**Fig. 3:**
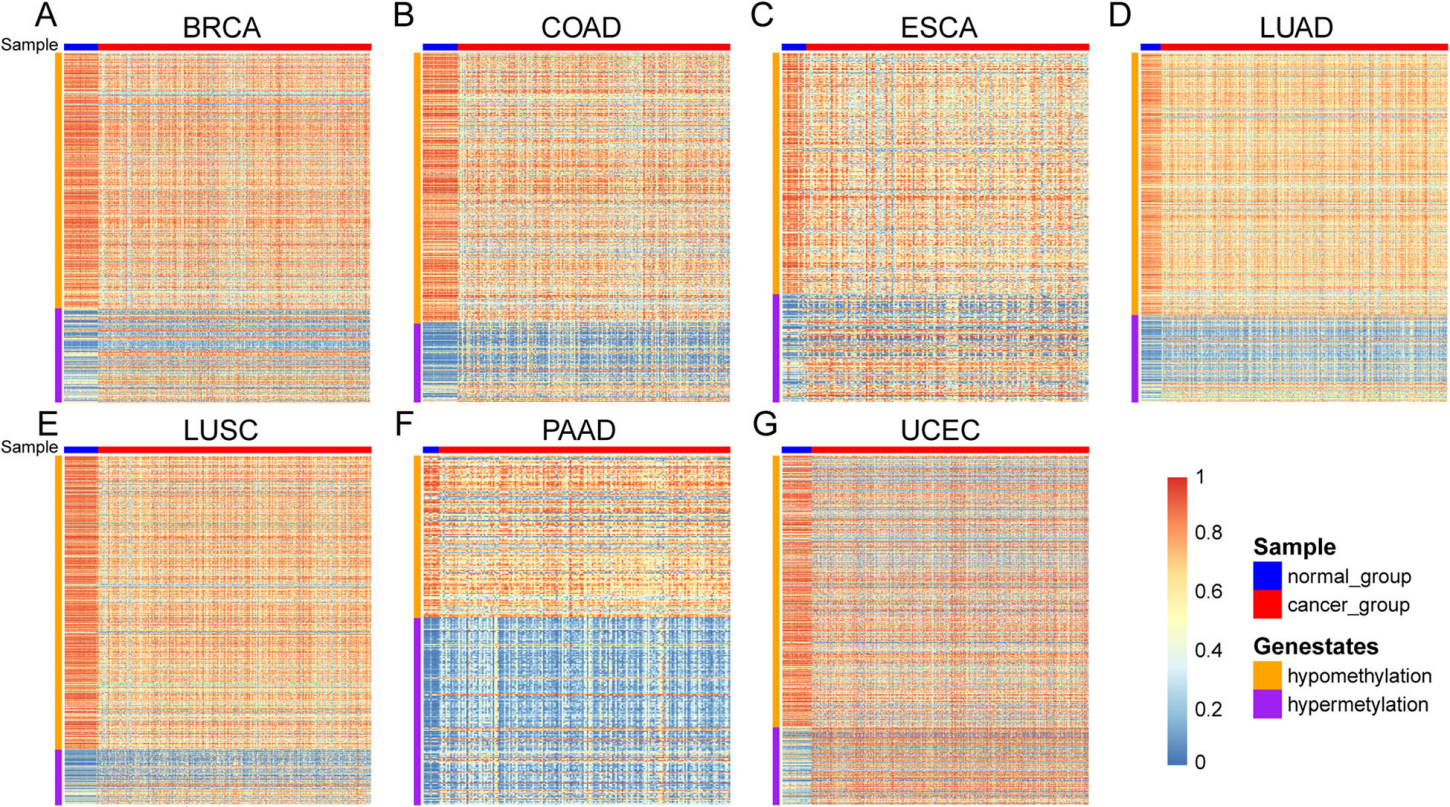
Heat map of differentially methylated genes in seven cancers. **a** Heat map of differentially methylated genes in BRCA. **b** Heat map of differentially methylated genes in COAD. **c** Heat map of differentially methylated genes in ESCA. **d** Heat map of differentially methylated genes in LUAD. **e** Heat map of differentially methylated genes in LUSC. **f** Heat map of differentially methylated genes in PAAD. **g** Heat map of differentially methylated genes in UCEC

All those differentially methylated genes are shown in Additional file 1: Figure S1, which indicates the great heterogeneity of differential methylation markers among the cancer types. Besides, we also use heat map to display the methylation level of differentially methylated genes in cancer samples and adjacent control samples (Fig. 3). We utilize the function pheatmap() in pheatmap package of R to create these graphs. It is from those graphs that we can see that each and every one of the differentially methylated genes of all the cancer types is able to separate cancer samples and adjacent control samples clearly.

### Heterogeneity of pathways and biological functions differentially methylated genes involved

**Fig. 4:**
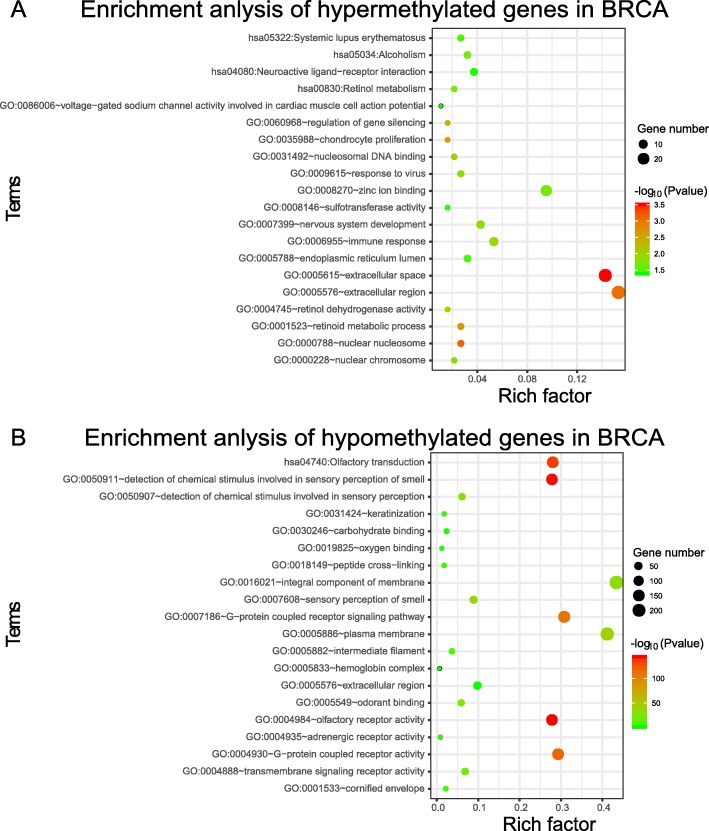
The enrichment analysis of differential methylated genes in BRCA. **a** The enrichment analysis of hypermethylated genes in BRCA. **b** The enrichment analysis of hypomethylated genes in BRCA

From the result of enrichment, we can see that differentially methylated genes in every cancer type are involved in various biological pathways and functions (Additional file 2: Figure S2, Additional file 3: Figure S3, Additional file 4: Figure S4, Additional file 5: Figure S5, Additional file 6: Figure S6, Additional file 7: Figure S7, Additional file 8: Figure S8). It was found that the most enriched gene ontology and KEGG pathways of these seven cancers are olfactory receptor activity, G-protein coupled receptor activity, odorant binding and Olfactory transduction, which have been reported to have association with cancers in previous studies [28–30]. At the same time, the distribution shown in Additional file 9: Figure S9 shows that the heterogeneity of biological pathways and functions enriched from differentially methylated genes among various cancer types are great. Specifically, 28 GO functions and 1 KEGG pathways are enriched from differentially methylated genes in two cancer types, 10 GO functions and 2 KEGG pathways are enriched from differentially methylated genes in three cancer types, 5 GO functions are enriched from differentially methylated genes in four cancer types, 2 GO functions are enriched from differentially methylated genes in five cancer types, 6 GO functions are enriched from differentially methylated genes in 6 cancer types, only 8 GO functions and 1 KEGG pathway are enriched from differentially methylated genes in all seven cancer types. The other 93 GO functions and 8 KEGG pathways are cancer specific, which shows that the heterogeneity of biological pathways and functions enriched from differentially methylated genes among various cancer types are great. Even within the same cancer type, differentially hypomethylated genes and hypermethylated could be involved in different pathways and functions. Enrichment pathways and top GO functions are shown in the graph (Fig. 4, Attached Additional file 10: Figure S10, Additional file 11: Figure S11, Additional file 12: Figure S12, Additional file 13: Figure S13, Additional file 14: Figure S14, Additional file 15: Figure S15).

### Identification and functional analysis of key genes in correlation network

We get 48,816 pairs of gene pairs whose DNA methylation levels are of strong correlation evidently, there are 7345 pairs in BRCA, 5477 in COAD, 5074 in ESCA, 24818 in LUAD, 4587 in LUSC, 9538 in PAAD, 1488 in UCEC.

**Fig. 5.**
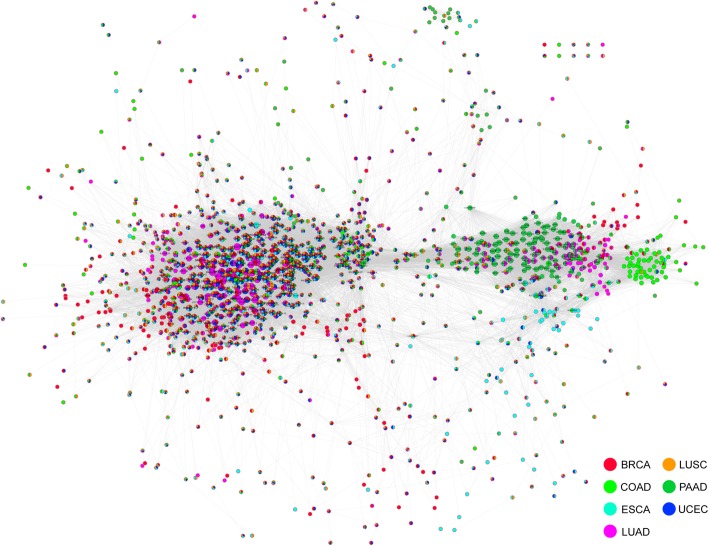
DNA methylation correlation network of differentially methylated genes. The nodes in network represent genes, and the edges represent a strong correlation between the two genes. The nodes marked as colors in the legend represent differential methylation of the gene in the cancer type, and a node with multiple color annotations indicates that the gene is differentially methylated in various cancers

The net contains a total number of 48,816 edges (Fig. 5). To assess biological significance of the pathway network, topological properties of the network is studied, the average degree of the nodes is 70.953 and the average clustering coefficient is 0.597, and above all, the degree of the network obeys power law distribution (Additional file 16: Figure S16), which indicates that this network conforms to the characteristics of scale-free biomolecular networks, that is, most of the nodes in the net have small degrees, only a small number of nodes have large degrees.

According to the degree ranking of the nodes, the first 274 genes are selected, with a maximal degree of 342 and a minimal degree of 137.Then, we discuss the function of those 274 genes, DAVID online bioinformatics tools are used in the enrichment analysis of the pathways and functions involved those genes. The result is visualized using EnrichmentMap function in Cytoscape (Additional file 17: Figure S17A). We can learn from the graph that these genes are significantly enriched in the biological processes related to G-protein-coupled receptor activity and signal pathway, ion channel-related biological processes and the regulation of cell proliferation and differentiation.

### Identification and analysis of key genes in KEGG pathway network

We obtain 6120 pairs of gene interactions in BRCA, 6934 in COAD, 4550 in ESCA, 5329 in LUAD, 6968 in LUSC, 2934 in PAAD, 7996 in UCEC.

**Fig. 6.**
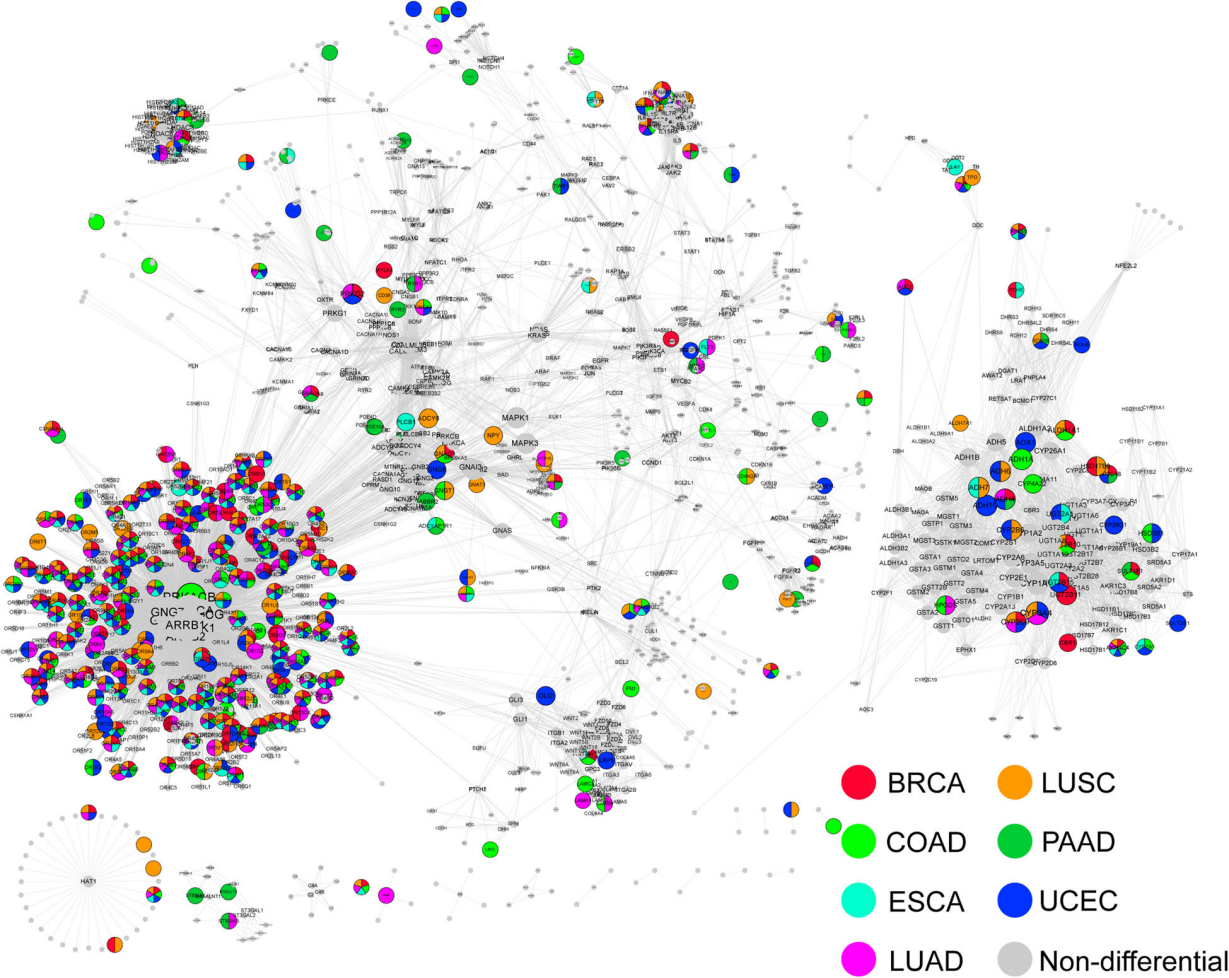
The KEGG pathway network. The nodes in network represent genes, and the edges represent the interaction of the two genes in the pathways. The nodes marked as colors in the legend represent differential methylation of the gene in the cancer type or a non-differentiated methylation gene obtained from the pathway. C. Enrichment analysis of prognostic marker candidate gene sets

The network of KEGG pathway is built (Fig. 6, Cytoscape 2.8.2 [17] (http://www.cytoscape.org/). The nodes in the network represent the genes in the pathways enriched by the differentially methylated genes in each type of cancer, and the edges represent the interaction between the two genes in the pathways. The colored nodes represent the gene is differentially methylated for this type of cancer, the gray nodes represent the non-differentially methylated genes extracted from the pathways but the genes that interact with differentially methylated genes. The size of the nodes is marked by the degree of the node, but the colored nodes are larger because different colors are required to be displayed. There are 1628 nodes and 12,765 edges in the network (Fig. 6). To assess biological significance of the pathway network, topological properties of the network is studied, the average degree of the nodes is 15.682 and the average clustering coefficient is 0.131, and above all, the degree of the network obeys power law distribution (Additional file 18: Figure S18), which indicates that this network conforms to the characteristics of scale-free biomolecular networks, that is, most of the nodes in the net have small degrees, only a small number of nodes have large degrees.

325 genes are selected with a maximal degree of 510 and a minimal degree of 18. Among those genes, 44 are genes differentially methylated in cancers, 281 are acquired from expansion of the pathways.

We also have a discussion on the functions of those genes. DAVID online bioinformatics tools are used to conduct an enrichment analysis on the pathways and functions in which those genes are involve, the results are visualized using EnrichmentMap in Cytoscape (Additional file 17: Figure S17B). Only the most significant enrichment (FDR < 1E-30) entries are shown in the figure, nodes in the graph represent biological functions or pathways where genes are significantly enriched, and the thickness of edge represents the correlation between these functions and pathways, which are measured by the number of shared genes. We can learn from the graph that those genes are significantly enriched in cancer and multiple signaling pathways, as well as metabolic and biosynthetic pathways.

### Integration and functional analysis of cancer-specific prognostic candidate marker sets

In this study, we first obtained the key candidate genes in various cancer types at the epigenetic modification level by DNA methylation correlation between genes, and further obtained more candidate genes from the perspective of functional interaction by pathway enrichment analysis.. The candidate gene obtained by these two methods has only one intersection gene (ADCYAP1R1), which is a common differential methylation gene among three cancers, COAD, PAAD and ESCA. The screening of these two complementary modes avoids the omission of the marker gene, and the candidate marker genes obtained by the two methods are integrated together as a basis for screening and analysis of the next specific cancer type prognostic marker. This study only performed a prognostic efficacy analysis of differentially methylated genes in each cancer type, thus removing 281 genes from the pathway that interacted with the differential genes. Finally, 317 differentially methylated genes in these cancers were obtained as prognostic marker candidate gene set.

Functional analysis of these candidate gene sets revealed significant enrichment of genes in sensory organ-related biological processes, many drug metabolisms, and biological processes and pathways for multiple enzyme synthesis (Additional file 17: Figure S17C). Therefore, it is speculated that abnormalities in these genes may lead to dysregulation of related biological processes and pathways, thus inducing cancer.

### Identification and analysis of specific prognostic markers per cancer

After the process mentioned above, we described sample information from two datasets for each cancer type in detail in Table 3, and we identify, from the univariate COX regression model, 4 prognostic risk markers for BRCA, 14 for COAD, 10 for ESCA, 7 for LUAD, 5 for LUSC, 16 for PAAD and 31 for UCEC, clinical factors are included as well as gene methylation. You can find information in detail in the attached table below. In the further analysis of multivariate COX regression, in all seven types of cancer, 3 risk genes that independently affecting prognosis of patients are found in BRCA, 6 in CPAD, 5 in ESCA, 2 in LUAD, 3 in LUSC, 11 in PAAD and 19 in UCEC. You can find information in detail in Table 4.

**Table 3:** Clinical characteristics of patients in the training set and testing set

Cancer type	Set	Stage				Age		Follow-up time (month)		Survival status	
		I	II	III	IV	Mean ± SD	Range	Mean ± SD	Range	Alive	Dead
BRCA	Trainingset	70	224	105	11	56.98 ± 12.98	26–90	31.46 ± 35.58	0–197	174	369
	Testing set	68	228	106	11	57.08 ± 13.26	26–90	31.3 ± 37.32	0–238	168	361
	P value	1^a^				0.93^b^		0.96^b^		0.95^a^	
COAD	Trainingset	29	67	52	28	64.84 ± 12.77	31–90	33.35 ± 32.72	1–151	40	115
	Testing set	27	61	46	25	64.86 ± 13.77	34–90	32.99 ± 27.68	2–143	40	115
	P value	1^a^				0.99^b^		0.92^b^		1^a^	
ESCA	Trainingset	20	50	40	16	62.52 ± 11.48	42–86	17.81 ± 14.96	1–69	39	54
	Testing set	22	52	39	16	62.31 ± 12.32	27–90	17.96 ± 18.07	1–124	39	54
	P value	0.99^a^				0.91^b^		0.95^b^		1^a^	
LUAD	Trainingset	129	57	41	12	65.1 ± 10.25	40–87	30.1 ± 30.96	1–236	86	151
	Testing set	126	60	37	13	65.16 ± 10.04	33–88	30.18 ± 30.13	1–242	86	148
	P value	0.96^a^				0.95^b^		0.98^b^		0.92^a^	
LUSC	Trainingset	92	70	31	5	67.6 ± 8.87	44–90	33.21 ± 31.36	1–157	83	109
	Testing set	85	70	30	4	67.56 ± 8.6	40–85	32.87 ± 32.26	1–177	81	108
	P value	0.98^a^				0.96^b^		0.92^b^		1^a^	
PAAD	Trainingset	11	77	3	4	64.73 ± 11.37	40–85	19.06 ± 14.41	1–77	50	43
	Testing set	12	76	4	4	64.84 ± 10.61	35–88	19.07 ± 16.96	1–92	50	43
	P value	1^a^				0.95^b^		1^b^		1^a^	
UCEC	Trainingset	131	20	52	13	64.21 ± 11.33	33–90	33.31 ± 28.82	1–229	38	180
	Testing set	133	23	49	11	64.22 ± 11.08	31–90	33.2 ± 28.08	1–189	38	178
	P value	0.92^a^				1^b^		0.97^b^		1^a^	

^a^Represents the p value calculated by Fisher’s exact test

^b^Represents the p value calculated by T test

**Table 4:** Results of multivariate COX regression analysis

Cancer	Prognostic marker	*β*	*P*	*HR*	Lower 95% CI	Upper 95% CI
BRCA	AKR1C4	−2.9373	0.0023	0.0530	0.0080	0.3491
	SNORD114.16	−2.8802	0.0051	0.0561	0.0075	0.4215
	SULT1E1	−3.3983	0.0133	0.0334	0.0023	0.4922
COAD	CCL4	−3.2497	0.0211	0.0388	0.0024	0.6143
	DEFB116	−5.1774	0.0007	0.0056	0.0003	0.1122
	MIR519C	−3.9175	0.0310	0.0199	0.0006	0.6989
	OR52E8	−3.8451	0.0117	0.0214	0.0011	0.4249
	SNORD113.5	−2.6062	0.0125	0.0738	0.0095	0.5708
	TRYX3	−2.3241	0.0255	0.0979	0.0127	0.7521
ESCA	ADCYAP1R1	3.2791	0.0027	26.5511	3.1161	226.2333
	CACNA2D3	2.4678	0.0166	11.7969	1.5667	88.8267
	KCNH5	1.8203	0.0361	6.1739	1.1256	33.8644
	SMO	2.2041	0.0228	9.0621	1.3597	60.3954
	TMEM132E	2.3834	0.0104	10.8415	1.7503	67.1527
LUAD	IL23R	2.2732	0.0192	9.7106	1.4478	65.1314
	TCP10L2	−2.1018	0.0254	0.1222	0.0194	0.7716
LUSC	OR6M1	1.8020	0.0263	6.0618	1.2370	29.7058
	REXO1L2P	−3.7316	0.0006	0.0240	0.0028	0.2027
	ZNF80	−1.8843	0.0148	0.1519	0.0334	0.6916
PAAD	ARL14	−3.2526	0.0023	0.0387	0.0048	0.3144
	DMRT1	2.9253	0.0109	18.6400	1.9621	177.0821
	KCNA1	2.6116	0.0075	13.6202	2.0105	92.2723
	KCNA5	3.0587	0.0345	21.3003	1.2494	363.1326
	KCNC1	5.1925	0.0095	179.9259	3.5557	9104.5993
	LOC641518	2.6193	0.0470	13.7262	1.0353	181.9858
	OR56A3	−3.2783	0.0131	0.0377	0.0028	0.5026
	PEX5L	3.0803	0.0139	21.7649	1.8721	253.0407
	SNORD114.29	−2.6840	0.0073	0.0683	0.0096	0.4860
	SOX14	4.1737	0.0112	64.9521	2.5839	1632.7153
	SULT1E1	−3.2091	0.0128	0.0404	0.0032	0.5049
UCEC	CNTN4	−1.4338	0.0398	0.2384	0.0608	0.9353
	IFNA7	2.1181	0.0124	8.3157	1.5815	43.7250
	IFNA8	2.0898	0.0085	8.0836	1.7051	38.3225
	MIR300	1.6945	0.0362	5.4441	1.1153	26.5741
	OR10AG1	−1.2951	0.0392	0.2739	0.0799	0.9382
	OR14C36	−1.4089	0.0311	0.2444	0.0679	0.8800
	OR1G1	1.7378	0.0397	5.6847	1.0849	29.7864
	OR2T10	−1.4348	0.0322	0.2382	0.0641	0.8855
	OR2T29	−1.6168	0.0192	0.1985	0.0513	0.7684
	OR2T5	−1.7935	0.0138	0.1664	0.0399	0.6933
	OR4A47	−1.5634	0.0352	0.2094	0.0489	0.8972
	OR5I1	−2.3936	0.0067	0.0913	0.0162	0.5153
	OR8H2	−1.9729	0.0141	0.1390	0.0288	0.6714
	OR8H3	−2.0804	0.0130	0.1249	0.0242	0.6450
	OR8K3	−2.1031	0.0008	0.1221	0.0356	0.4190
	OR9G4	−1.6672	0.0088	0.1888	0.0542	0.6573
	SNORD113.5	1.4484	0.0397	4.2564	1.0705	16.9240
	SNORD114.16	1.7309	0.0104	5.6459	1.5029	21.2101
	UGT2B15	1.5478	0.0338	4.7012	1.1257	19.6330

**Fig. 7.**
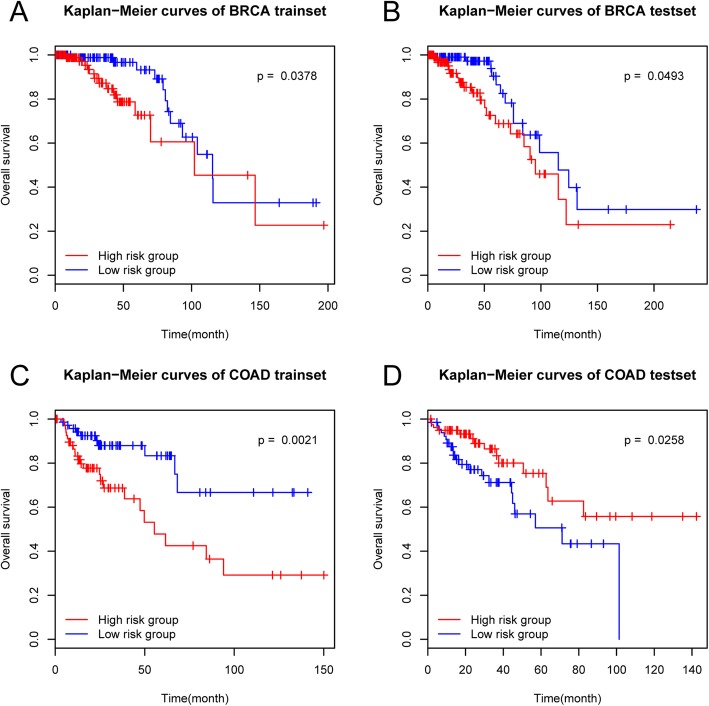
Kaplan-Meier survival curve. **a** Survival curve of BRCA training set. **b** Survival curve of BRCA test set. **c** Survival curve of COAD training set. **d**. Survival curve of COAD test set

Survival analysis of the two groups of patients of each type of cancer shows that there are significant differences in survival between the two risk groups in all types of cancer (Fig. 7, attached Additional file 19: Figure S19). Further validation based on the reserved test set using the method stated above shows that there are significant differences in survival between the two groups in all the seven types of cancer except ESCA whose *p* value of significance is 0.0563 (higher than 0.05) (Fig. 7 attached Additional file 19: Figure S19). Although the significance of ESCA does not reach below 0.05, as we can tell from the figure, the two groups of patients can be separated using the prognostic marker genes sifted out. This suggests that the prognostic markers screened out in this study are reliable and can be used to distinguish the high and low risk of patients. And it’s also worth noting that, prognostic markers, in most types of cancer, are specific to this type of cancer. A few exceptions are the one common prognostic marker in BRCA and UCEC (SNORD114.16), SULT1E1 in BRCA and PAAD, SNORD113.5 in COAD and UCEC. SULT1E1 is a protective factor in both BRCA and PAAD, however, The other two markers play opposite roles in the two types of cancer (risk factor and protective factor).

After looking through papers, only 4 genes of these prognostic markers have been verified to be relavant with according cancers, including CCL4 [31, 32] in COAD, CACNA2D3 [33, 34] and SMO [35–37] in ESCA, and IL23R [38] in LUAD. Other genes have not been tested to be efficient in treating cancer, which may be potential targets for scientists and doctors to further research on them.

## Discussion

The heterogeneity of cancers is one of the reasons why cancers are so hard to be cured clinically, therefore, molecular analysis of the mechanism of cancer heterogeneity and screening of cancer-specific diagnostic and prognostic molecular markers are of great importance for clinical treatment. In addition to genetic mutations, DNA methylation is an important epigenetic alteration that can modify gene expression and is commonly perturbed in cancers [39]. So far, DNA methylation is proposed as a molecular biomarker for cancer detection [40] but also as a biomarker for prediction and stratification of patients with risk of distinct clinical outcome and response to therapies [41], which are found abnormal in the early stage of cancer generation which is a stable marker in cancers. It is a severer change in that it affects the transcriptional regulation of genes, which makes it a potentially important marker for early detection, precise treatment and prognosis assessment of cancer. In cancer detection, DNA methylation also has several advantages over somatic mutation analysis, such as high clinical sensitivity and dynamic range. Moreover, the change of DNA methylation pattern is one of the first detectable tumor-specific changes associated with tumorigenesis. Therefore, it is an important research direction to interpret the heterogeneity of cancer from the perspective of epigenetic abnormality.

Yang et al. provides a comprehensive investigation and reveals meaningful cancer common and specific DNA methylation patterns, contributing to a deeper understanding of pan-cancer studies [42]. They discovered a potential tumorigenesis mechanism that involved of three pan-cancer differentially methylated CpG sites (PDMCs) and 62 PDMCs that are significantly associated with patient survival. They also found that cancer-specific DMCs are enriched in known cancer genes and cell-type-specific super-enhancers.

We also conducted a research on pan-cancer analysis from epigenetic perspective. Compared to the study conducted by Yang at al, we first performed a differential methylation analysis of genes (DMGs) and aimed to find reliable prognostic markers for each cancer from gene levels, and made a supplementation of their survival analysis. In this study, the heterogeneity of DNA methylation markers among cancers is discussed in detail by using the large sample DNA methylation data of seven cancers in TCGA database detected by the open available HM450K chip platform. Differential methylation analysis identifies specific and common tumor markers in each type of cancer, which provides more potential targets for cancer diagnosis and experimental researchers. These cancer type-specific tumor markers are also involved in different biological functions and pathways. In the next step, through using two biological molecular networks, DNA methylation correlation network and KEGG pathway network, the marker sets are further optimized and integrated from the perspective of correlation and functional interaction. At last, the specific prognostic markers for each type of cancer are screened out by using the establishment of prognostic model. These markers can classify the risk of patients ideally, and are verified in the test set. The searching of prognostic markers for cancer provides important reference for clinicians to monitor conditions of patients and to alter regimens of treatment in time.

## Conclusions

In this study, DNA methylation markers of only 7 cancer types in TCGA are screened out and analyzed, but the method in this study is also applicable to other cancer types. Also, though the preliminary verification of these markers is realized by the compute in this study, which lays a solid theoretical foundation for the reliability of these markers, further experimental confirmation is still a necessity to promote the process in which those molecular markers are put into clinical use.

## Supplementary information

**Additional file 1:** **Figure S1.** The numbers of differentially methylated genes in seven cancers.

**Additional file 2:** **Figure S2.** The enrichment analysis of all differential methylated genes in BRCA. The figure shows the enriched pathways and the top 17 GO terms.

**Additional file 3:** **Figure S3.** The enrichment analysis of all differential methylated genes in COAD. The figure shows the enriched pathways and the top 20 GO terms.

**Additional file 4:** **Figure S4.** The enrichment analysis of all differential methylated genes in ESCA. The figure shows the enriched pathways and the top 16 GO terms.

**Additional file 5:** **Figure S5.** The enrichment analysis of all differential methylated genes in LUAD. The figure shows the enriched pathways and the top 20 GO terms.

**Additional file 6:** **Figure S6.** The enrichment analysis of all differential methylated genes in LUSC. The figure shows the enriched pathways and the top 20 GO terms.

**Additional file 7:** **Figure S7.** The enrichment analysis of all differential methylated genes in PAAD. The figure shows the enriched pathways and the top 20 GO terms.

**Additional file 8:** **Figure S8.** The enrichment analysis of all differential methylated genes in UCEC. The figure shows the enriched pathways and the top 20 GO terms.

**Additional file 9:** **Figure S9.** The numbers of GO functions and KEGG pathways enriched by differentially methylated genes in seven cancers. A. The number of GO functions enriched by differential methylated genes in seven cancers. B. The number of KEGG pathways enriched by differentially methylated in seven cancers.

**Additional file 10:** **Figure S10.** The enrichment analysis of differential methylated genes in COAD. A. The enrichment analysis of hypermethylated genes in COAD. B. The enrichment analysis of hypomethylated genes in COAD.

**Additional file 11:** **Figure S11.** The enrichment analysis of differential methylated genes in ESCA. A. The enrichment analysis of hypermethylated genes in ESCA. B. The enrichment analysis of hypomethylated genes in ESCA.

**Additional file 12:** **Figure S12.** The enrichment analysis of differential methylated genes in LUAD. A. The enrichment analysis of hypermethylated genes in LUAD. B. The enrichment analysis of hypomethylated genes in LUAD.

**Additional file 13:** **Figure S13.** The enrichment analysis of differential methylated genes in LUSC. A. The enrichment analysis of hypermethylated genes in LUSC. B. The enrichment analysis of hypomethylated genes in LUSC.

**Additional file 14:** **Figure S14.** The enrichment analysis of differential methylated genes in PAAD. A. The enrichment analysis of hypermethylated genes in PAAD. B. The enrichment analysis of hypomethylated genes in PAAD.

**Additional file 15:** **Figure S15.** The enrichment analysis of differential methylated genes in UCEC. A. The enrichment analysis of hypermethylated genes in UCEC. B. The enrichment analysis of hypomethylated genes in UCEC.

**Additional file 16:** **Figure S16.** The node degree distribution of the DNA methylation correlation network.

**Additional file 17:** **Figure S17.** Enrichment analysis of key genes in DNA methylation network. A. Enrichment analysis of key genes in DNA methylation correlation network. B. Enrichment analysis of key genes in KEGG pathway network.

**Additional file 18:** **Figure S18.** The node degree distribution of the KEGG pathway network.

**Additional file 19:** **Figure S19.** Kaplan-Meier survival curve. A. Survival curve of ESCA training set. B. Survival curve of ESCA test set. C. Survival curve of LUAD training set. D. Survival curve of LUAD test set. E. Survival curve of LUSC training set. F. Survival curve of LUSC test set. G. Survival curve of PAAD training set. H. Survival curve of PAAD test set. I. Survival curve of UCEC training set. J. Survival curve of UCEC test set.

## Data Availability

All data analyzed in this study are from open data (freely available to anyone) at TCGA database: “https://xenabrowser.net/datapages/” and GEO dataset: “https://www.ncbi.nlm.nih.gov/geo/query/acc.cgi?acc=GPL13534”.

## Abbreviations

COAD: colon adenocarcinoma
ESCA: esophageal cancer
FDR: false discovery rate
GO: Gene Ontology
KEGG: Kyoto Encyclopedia of Genes and Genomes
LUAD: lung adenocarcinoma
LUSC: lung squamous cell carcinoma
MBDs: methyl-binding domain proteins
PAAD: pancreatic cancer
TCGA: The Cancer Genome Atlas
