# Supplementary figures and images for: An analysis about heterogeneity among cancers based on the DNA methylation patterns
Source: BMC Cancer. 2019 Dec 30;19:1259. doi: 10.1186/s12885-019-6455-x (PMC6937830; doi:10.1186/s12885-019-6455-x)

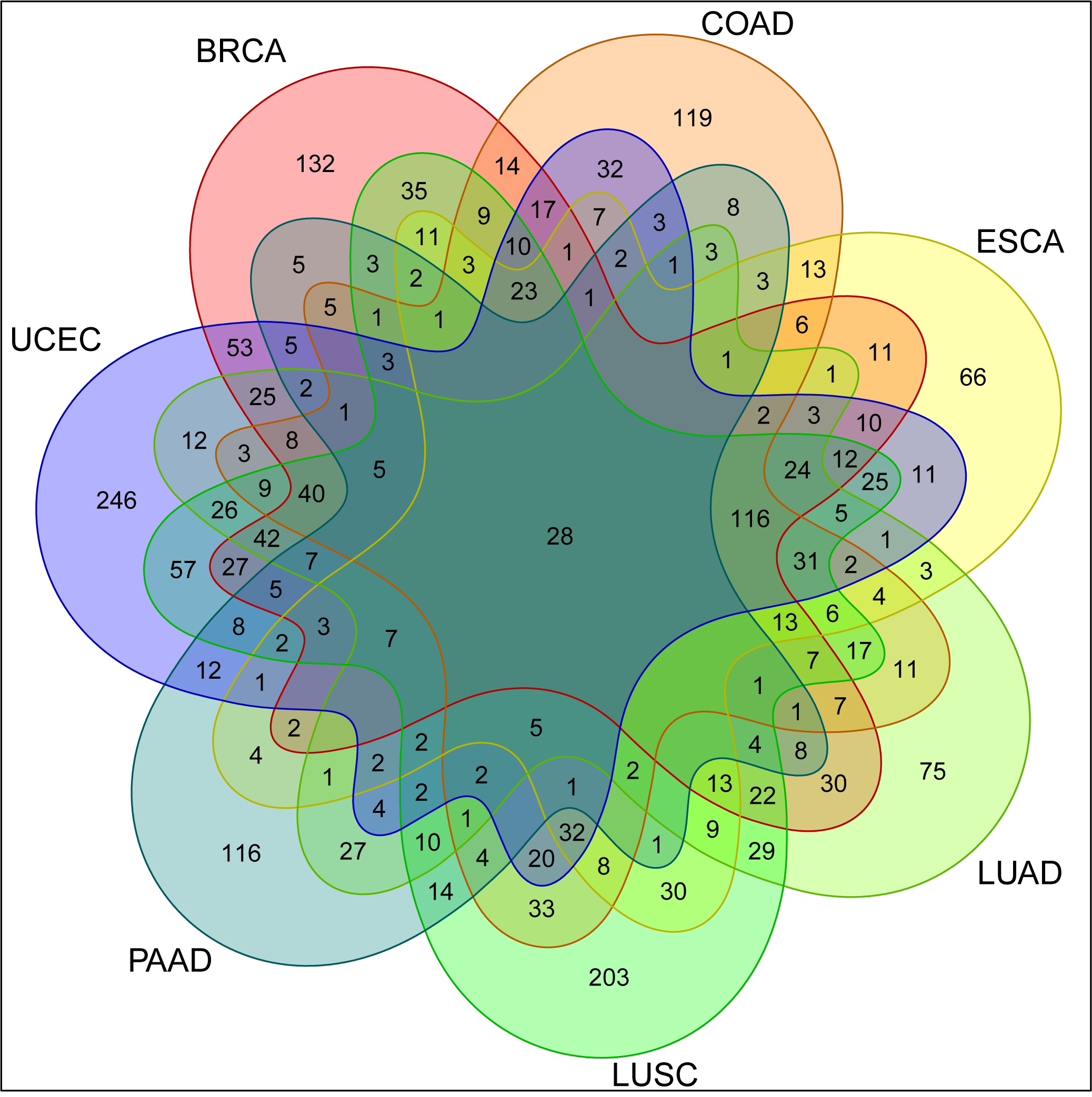

Supplement: Supplementary file 1 — Additional file 1: Figure S1. The numbers of differentially methylated genes in seven cancers. [file 12885_2019_6455_MOESM1_ESM.tif]

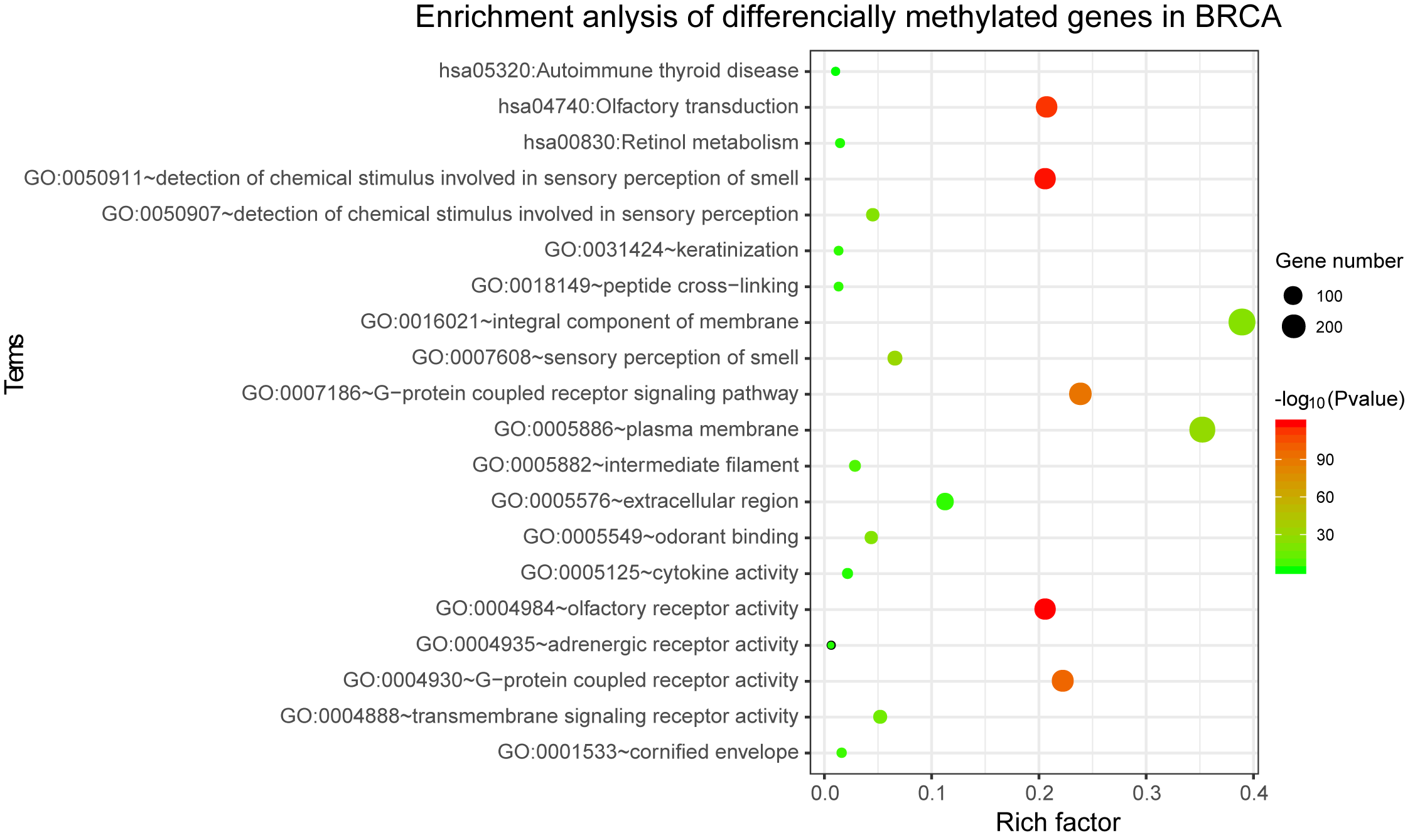

Supplement: Supplementary file 2 — Additional file 2: Figure S2. The enrichment analysis of all differential methylated genes in BRCA. The figure shows the enriched pathways and the top 17 GO terms. [file 12885_2019_6455_MOESM2_ESM.tif]

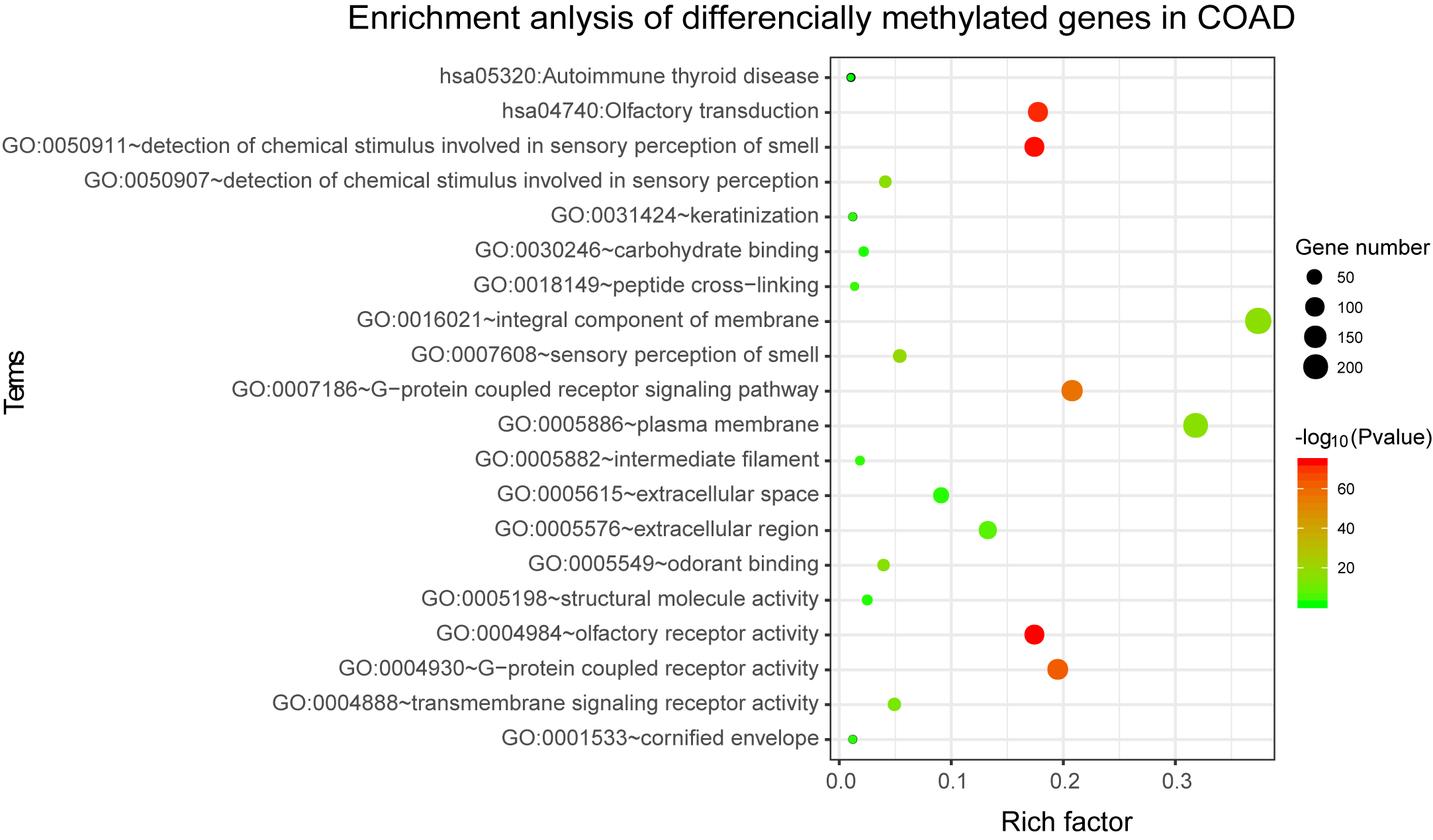

Supplement: Supplementary file 3 — Additional file 3: Figure S3. The enrichment analysis of all differential methylated genes in COAD. The figure shows the enriched pathways and the top 20 GO terms. [file 12885_2019_6455_MOESM3_ESM.tif]

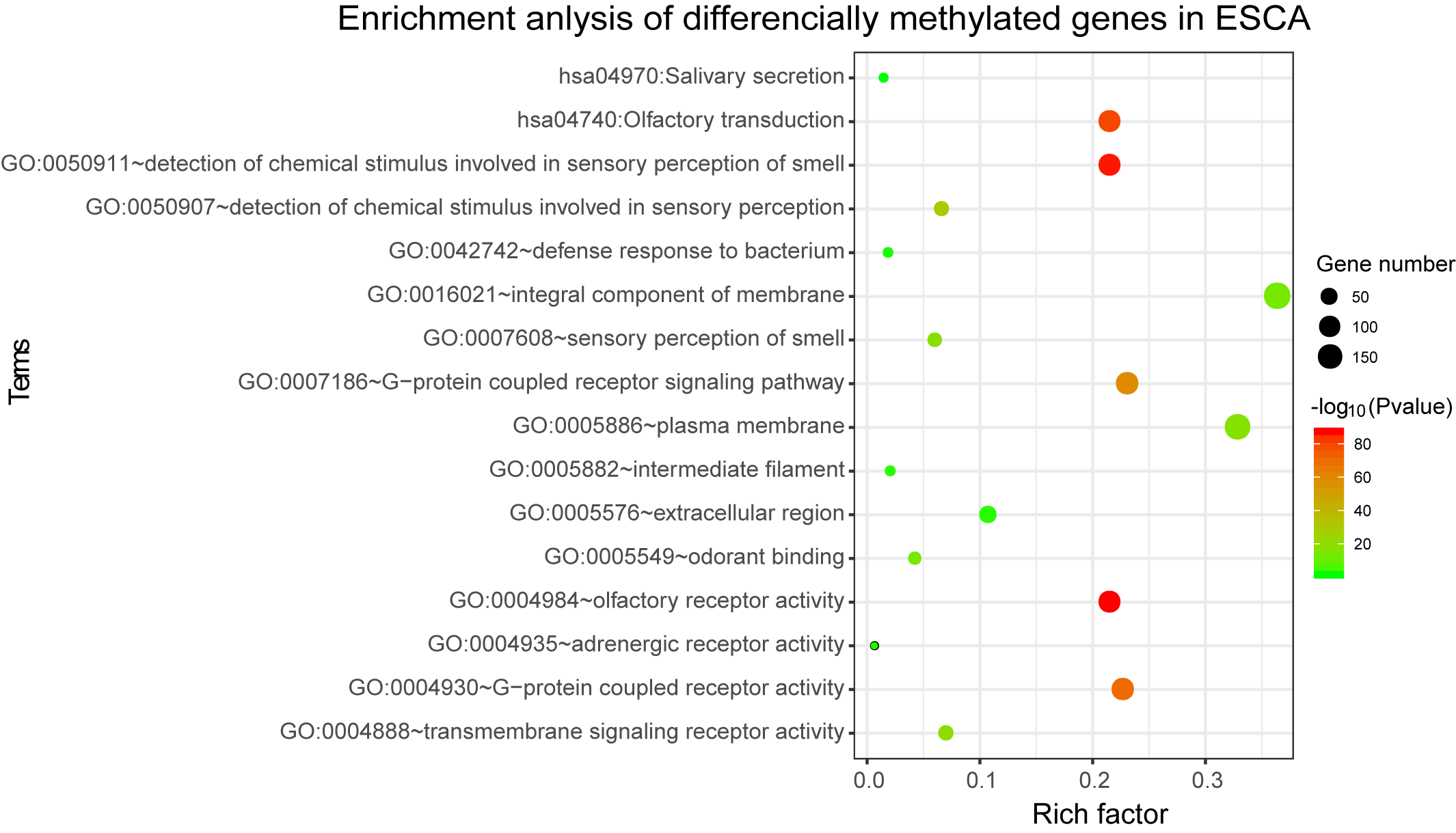

Supplement: Supplementary file 4 — Additional file 4: Figure S4. The enrichment analysis of all differential methylated genes in ESCA. The figure shows the enriched pathways and the top 16 GO terms. [file 12885_2019_6455_MOESM4_ESM.tif]

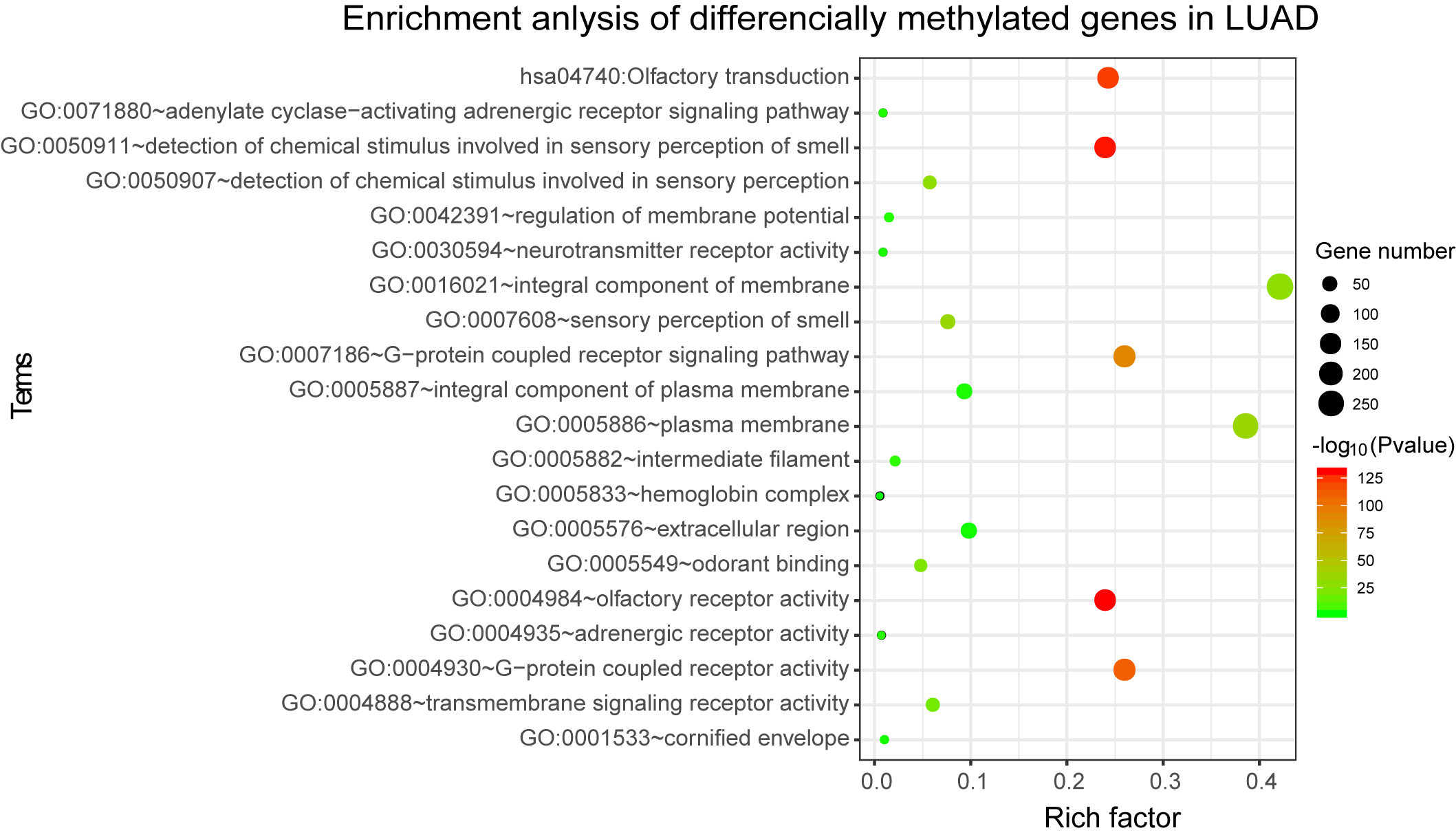

Supplement: Supplementary file 5 — Additional file 5: Figure S5. The enrichment analysis of all differential methylated genes in LUAD. The figure shows the enriched pathways and the top 20 GO terms. [file 12885_2019_6455_MOESM5_ESM.tif]

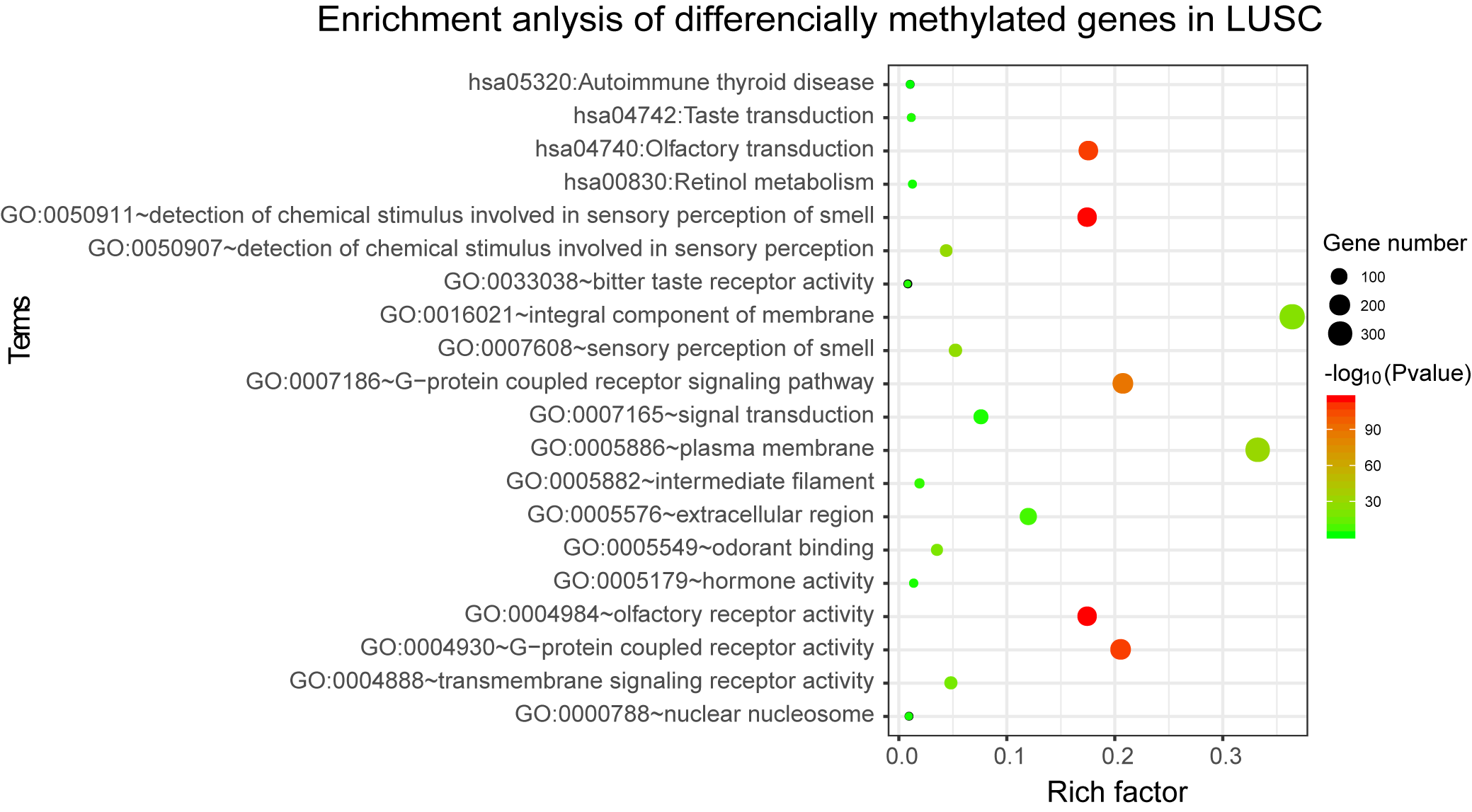

Supplement: Supplementary file 6 — Additional file 6: Figure S6. The enrichment analysis of all differential methylated genes in LUSC. The figure shows the enriched pathways and the top 20 GO terms. [file 12885_2019_6455_MOESM6_ESM.tif]

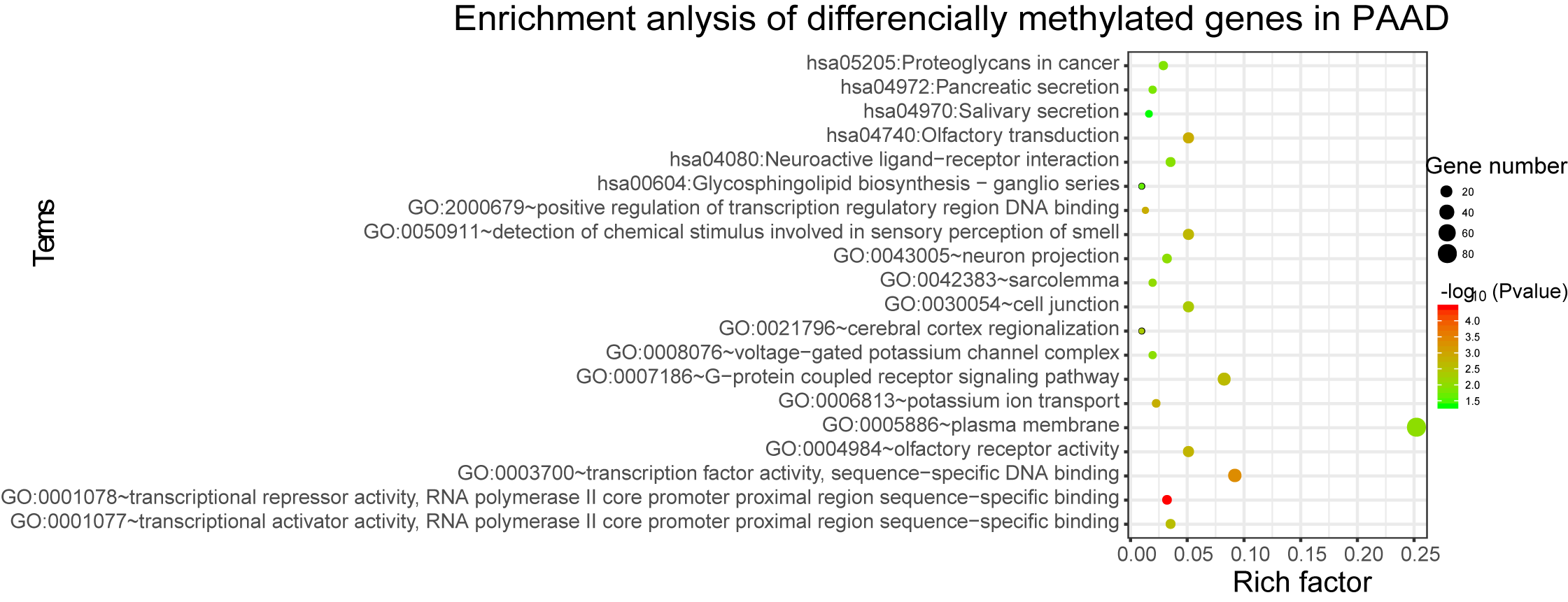

Supplement: Supplementary file 7 — Additional file 7: Figure S7. The enrichment analysis of all differential methylated genes in PAAD. The figure shows the enriched pathways and the top 20 GO terms. [file 12885_2019_6455_MOESM7_ESM.tif]

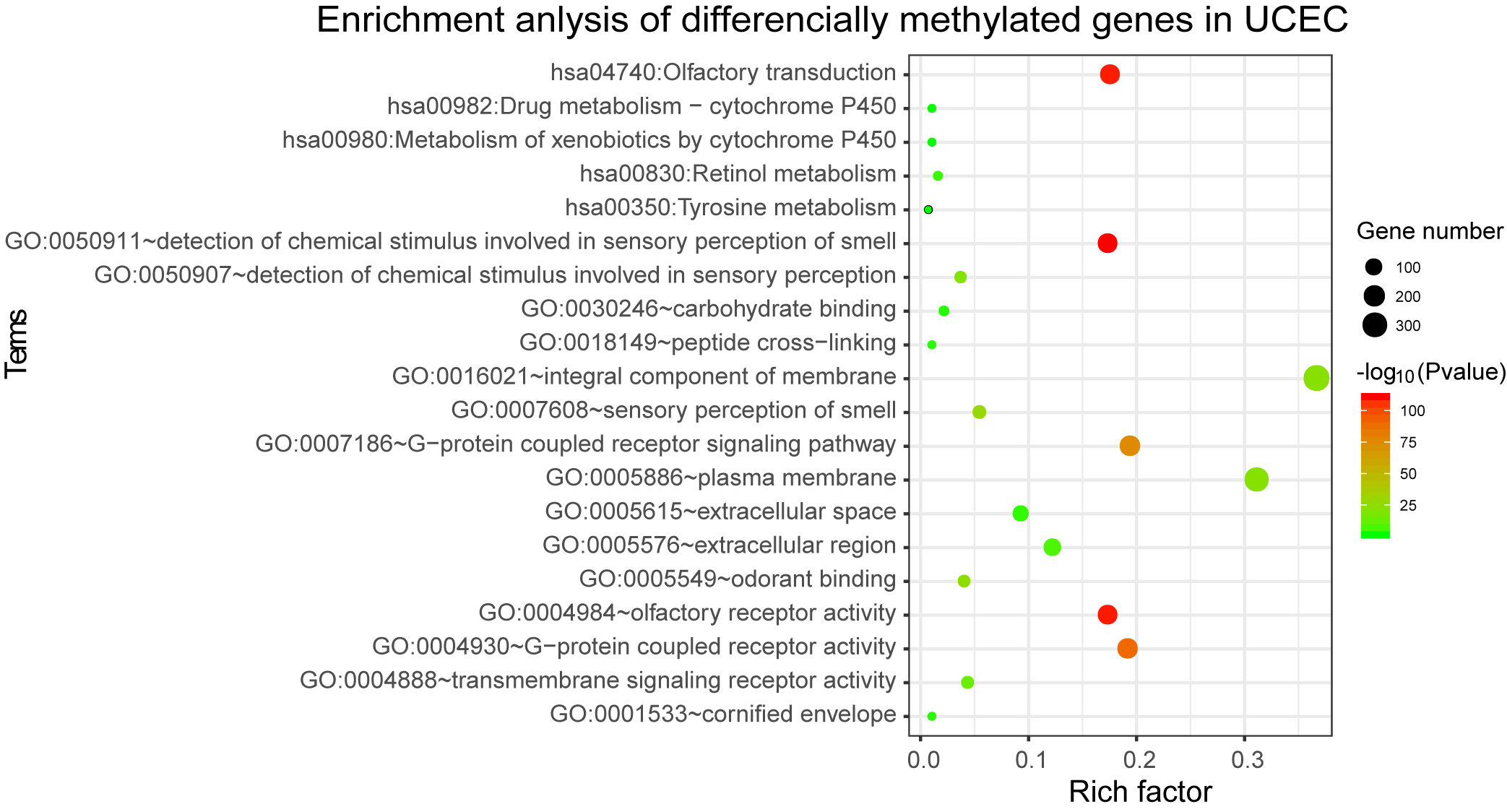

Supplement: Supplementary file 8 — Additional file 8: Figure S8. The enrichment analysis of all differential methylated genes in UCEC. The figure shows the enriched pathways and the top 20 GO terms. [file 12885_2019_6455_MOESM8_ESM.tif]

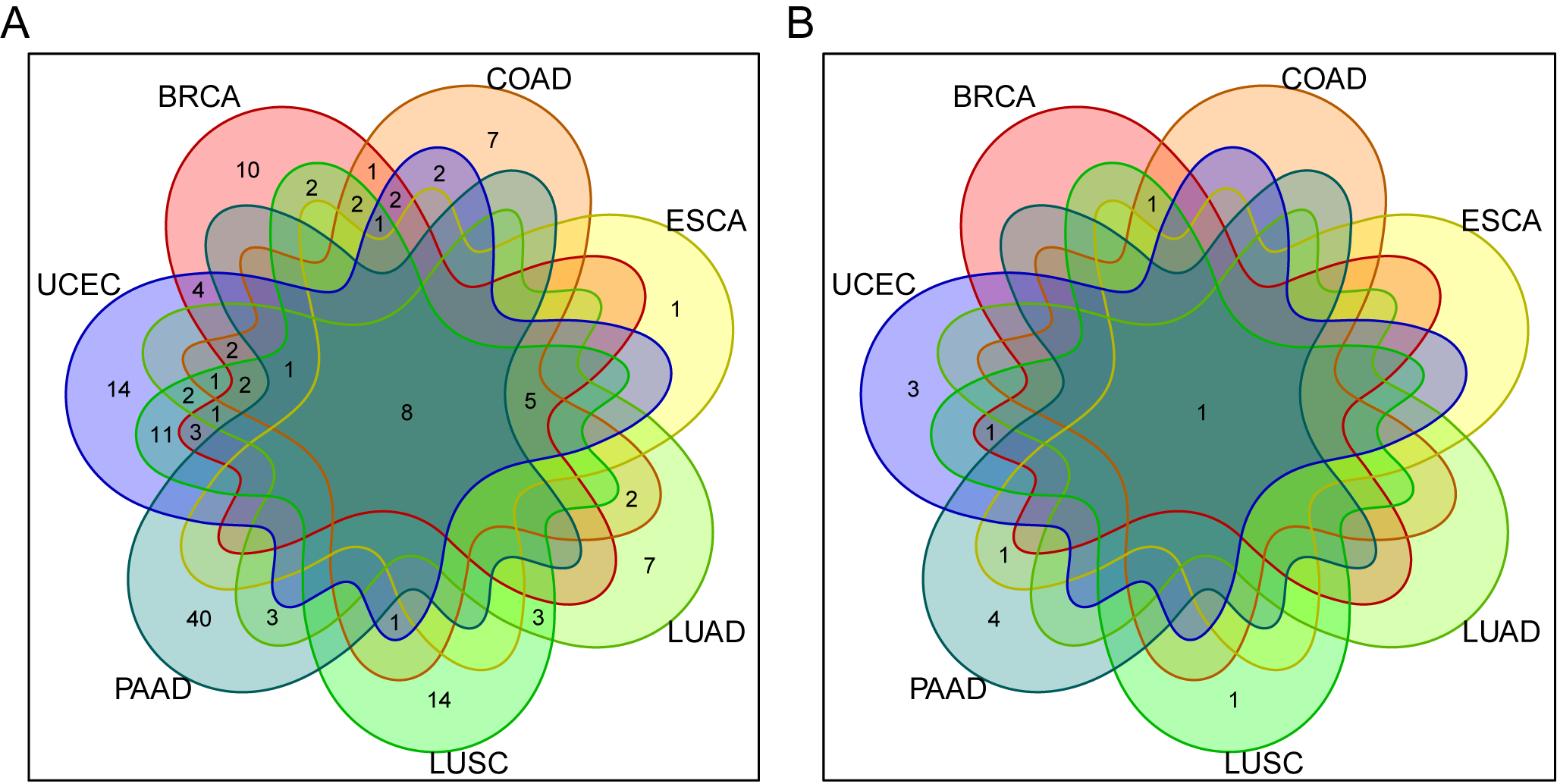

Supplement: Supplementary file 9 — Additional file 9: Figure S9. The numbers of GO functions and KEGG pathways enriched by differentially methylated genes in seven cancers. A. The number of GO functions enriched by differential methylated genes in seven cancers. B. The number of KEGG pathways enriched by differentially methylated in seven cancers. [file 12885_2019_6455_MOESM9_ESM.tif]

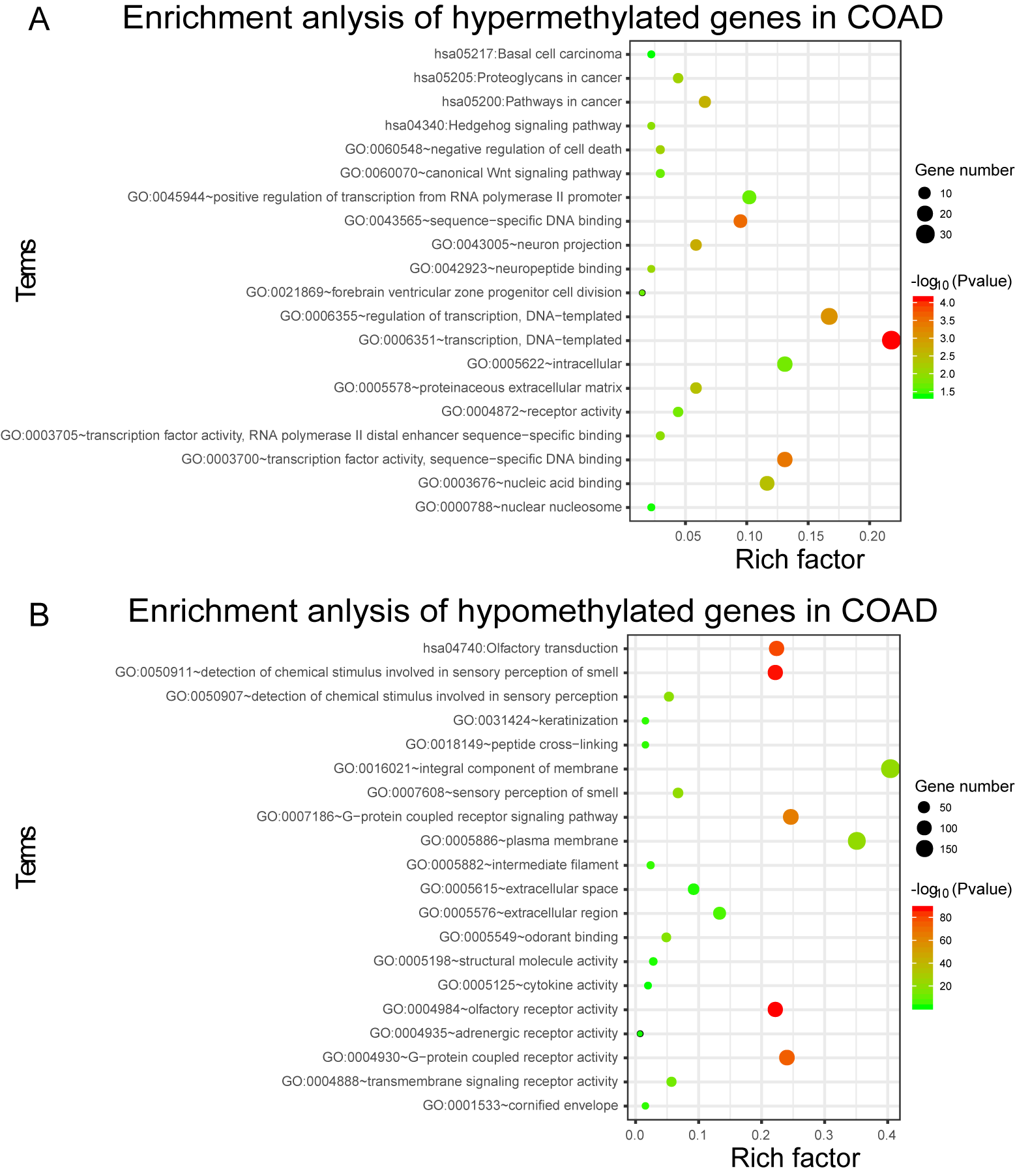

Supplement: Supplementary file 10 — Additional file 10: Figure S10. The enrichment analysis of differential methylated genes in COAD. A. The enrichment analysis of hypermethylated genes in COAD. B. The enrichment analysis of hypomethylated genes in COAD. [file 12885_2019_6455_MOESM10_ESM.tif]

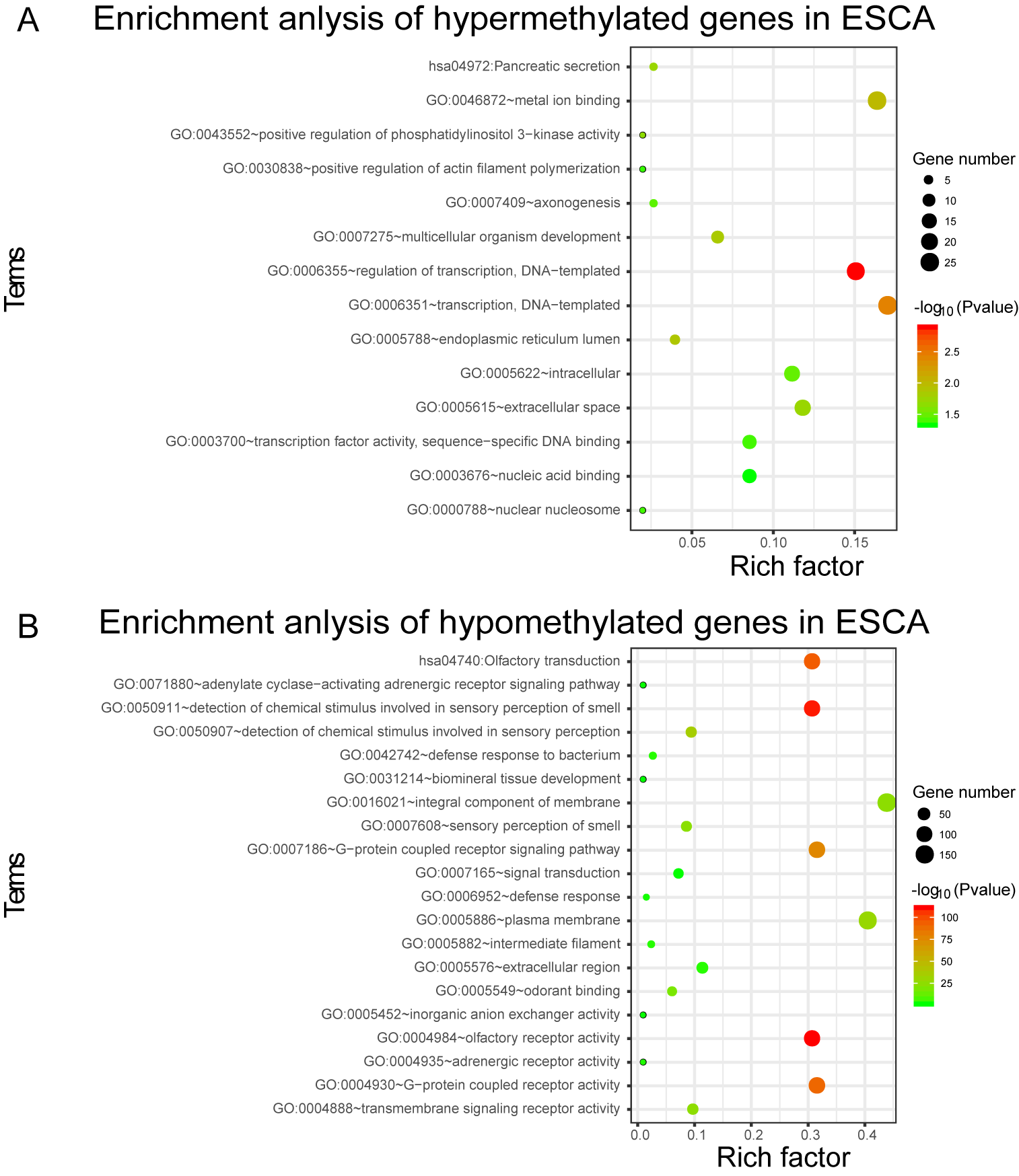

Supplement: Supplementary file 11 — Additional file 11: Figure S11. The enrichment analysis of differential methylated genes in ESCA. A. The enrichment analysis of hypermethylated genes in ESCA. B. The enrichment analysis of hypomethylated genes in ESCA. [file 12885_2019_6455_MOESM11_ESM.tif]

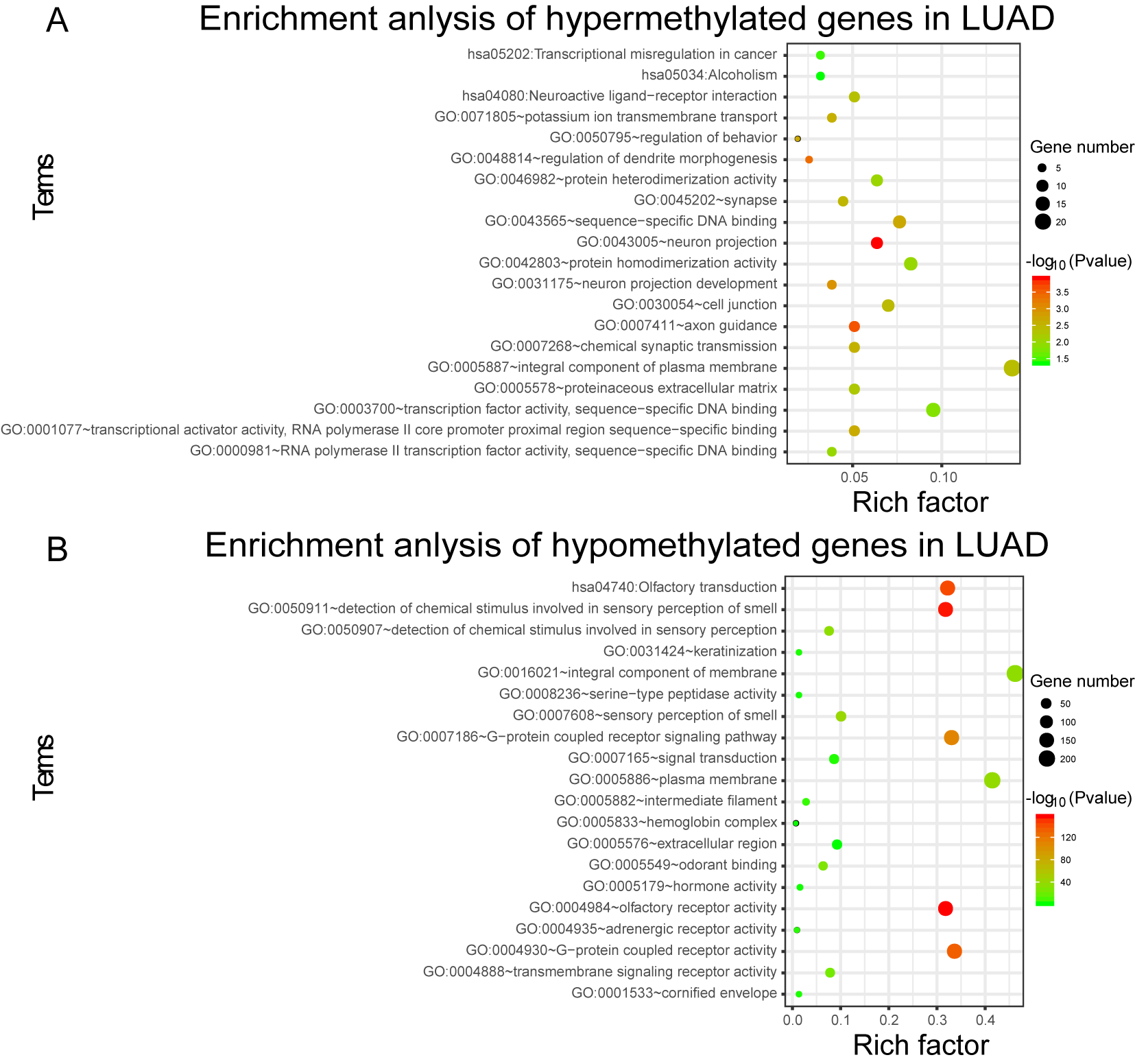

Supplement: Supplementary file 12 — Additional file 12: Figure S12. The enrichment analysis of differential methylated genes in LUAD. A. The enrichment analysis of hypermethylated genes in LUAD. B. The enrichment analysis of hypomethylated genes in LUAD. [file 12885_2019_6455_MOESM12_ESM.tif]

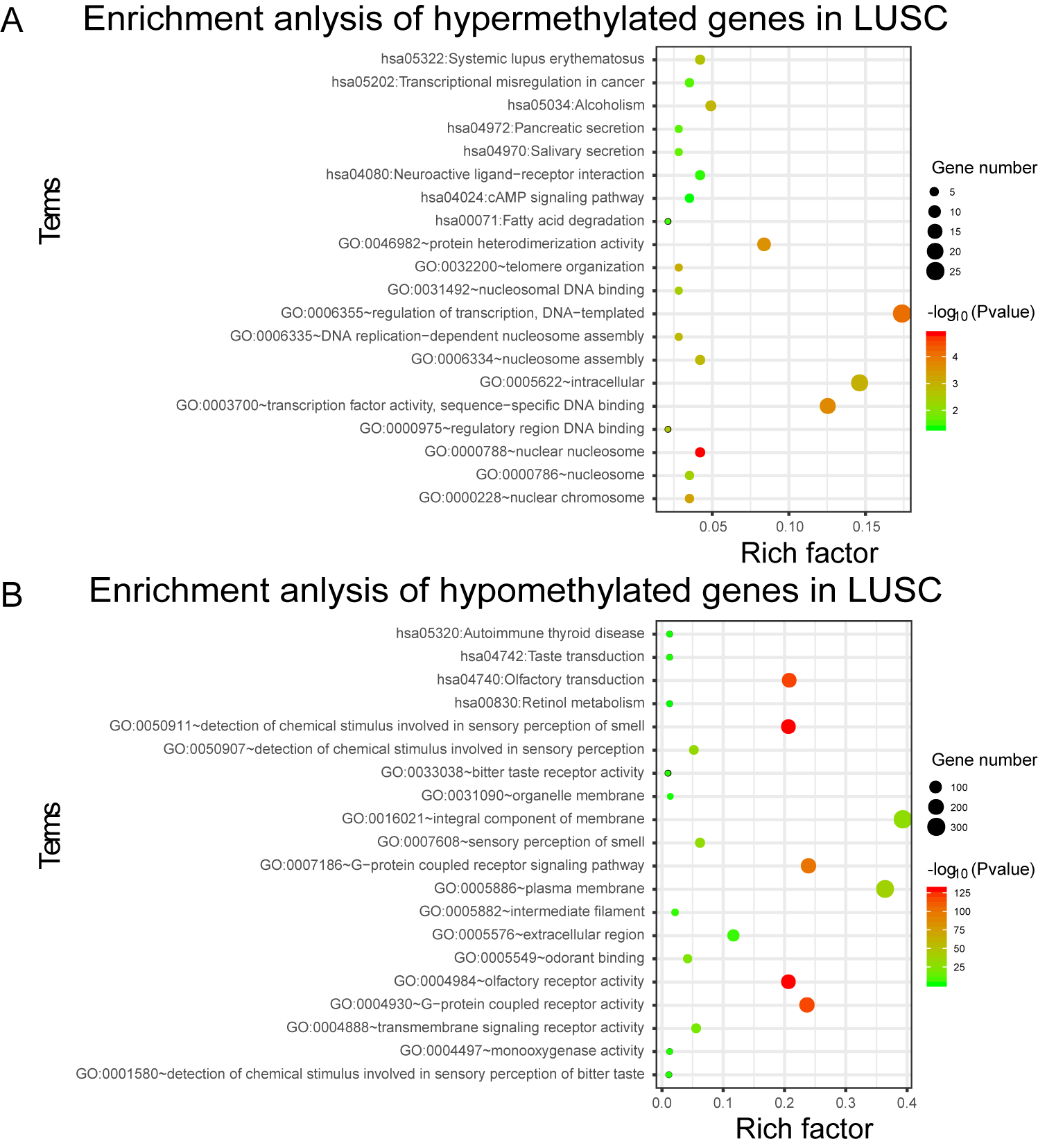

Supplement: Supplementary file 13 — Additional file 13: Figure S13. The enrichment analysis of differential methylated genes in LUSC. A. The enrichment analysis of hypermethylated genes in LUSC. B. The enrichment analysis of hypomethylated genes in LUSC. [file 12885_2019_6455_MOESM13_ESM.tif]

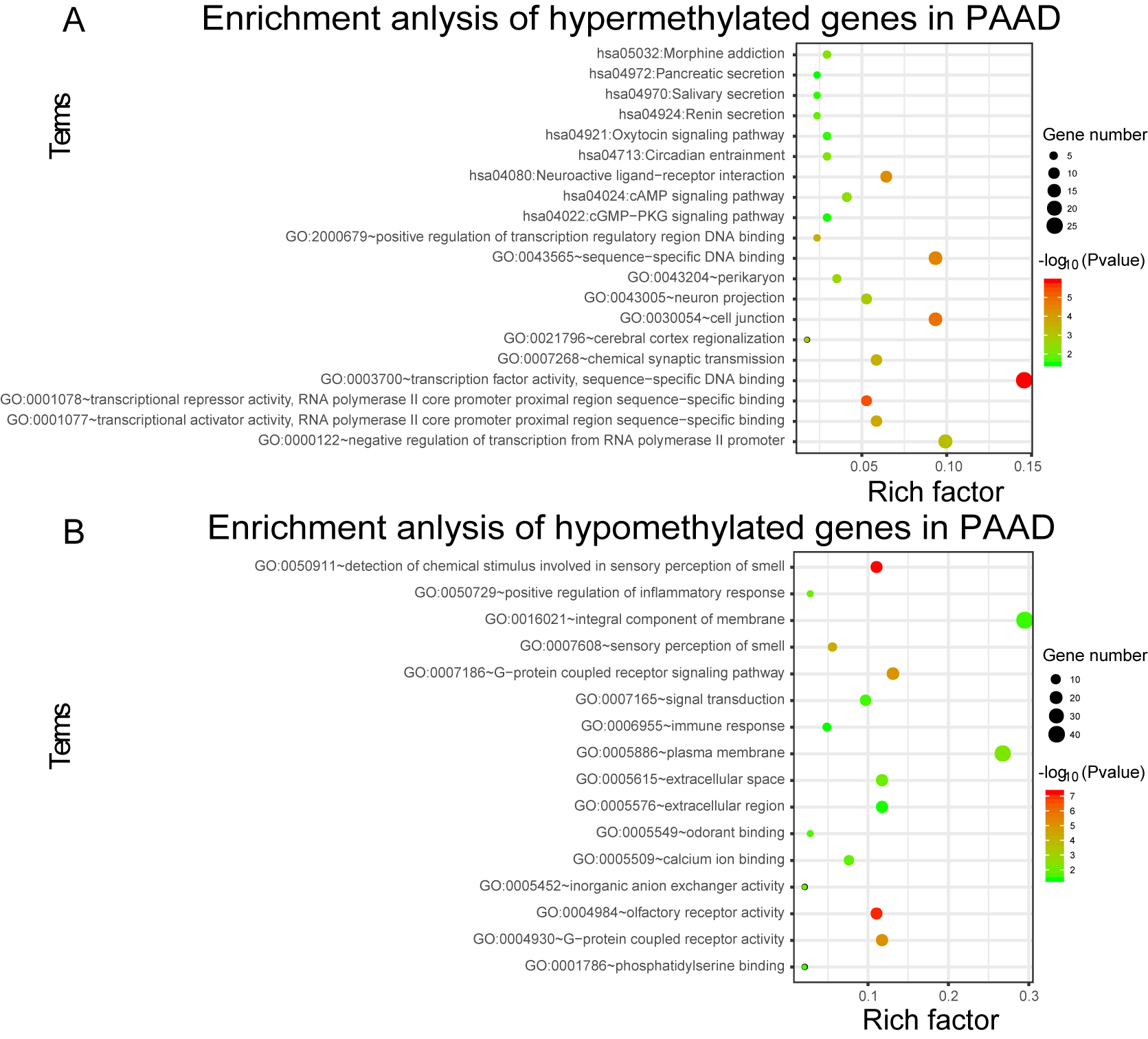

Supplement: Supplementary file 14 — Additional file 14: Figure S14. The enrichment analysis of differential methylated genes in PAAD. A. The enrichment analysis of hypermethylated genes in PAAD. B. The enrichment analysis of hypomethylated genes in PAAD. [file 12885_2019_6455_MOESM14_ESM.tif]

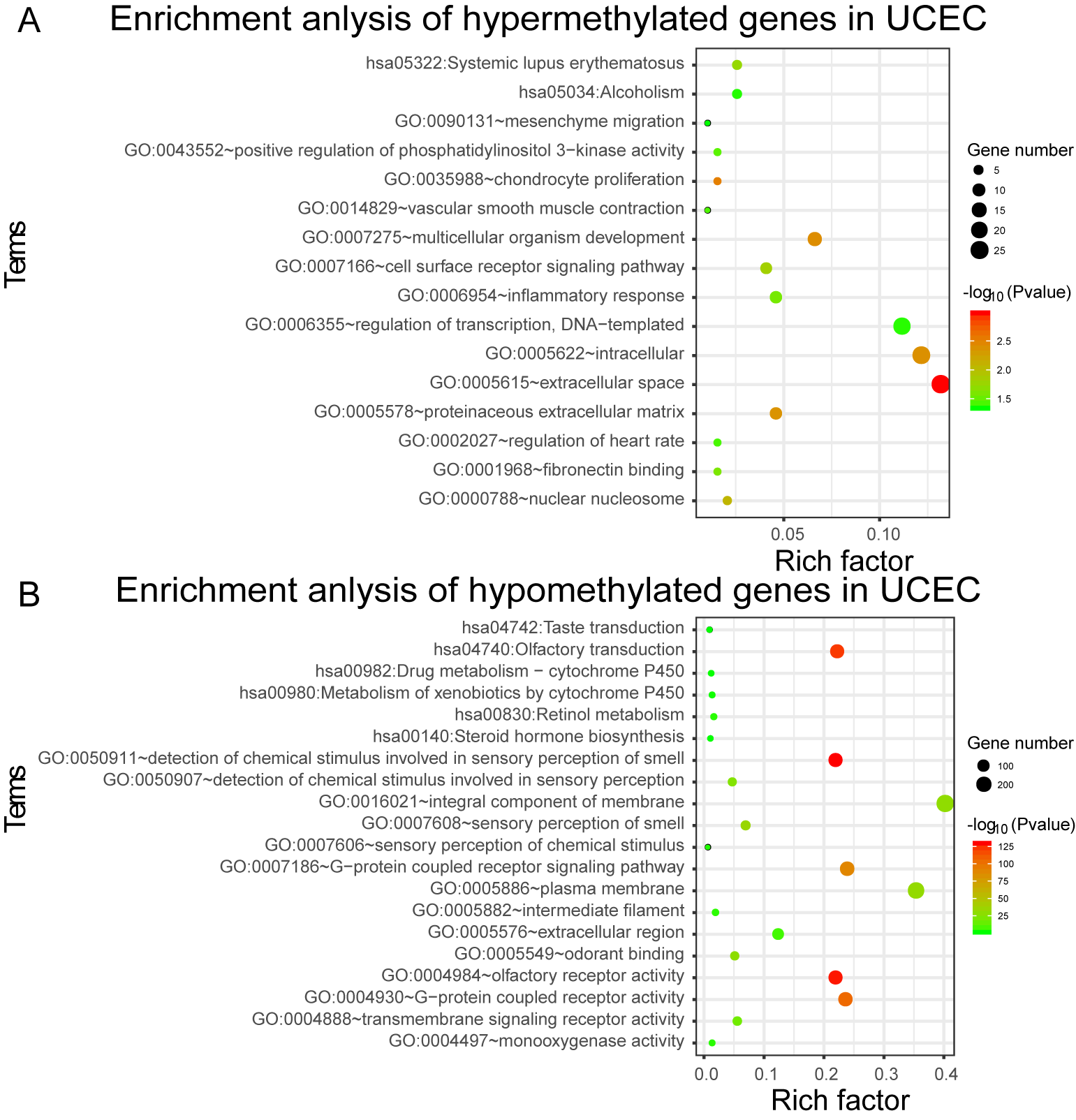

Supplement: Supplementary file 15 — Additional file 15: Figure S15. The enrichment analysis of differential methylated genes in UCEC. A. The enrichment analysis of hypermethylated genes in UCEC. B. The enrichment analysis of hypomethylated genes in UCEC. [file 12885_2019_6455_MOESM15_ESM.tif]

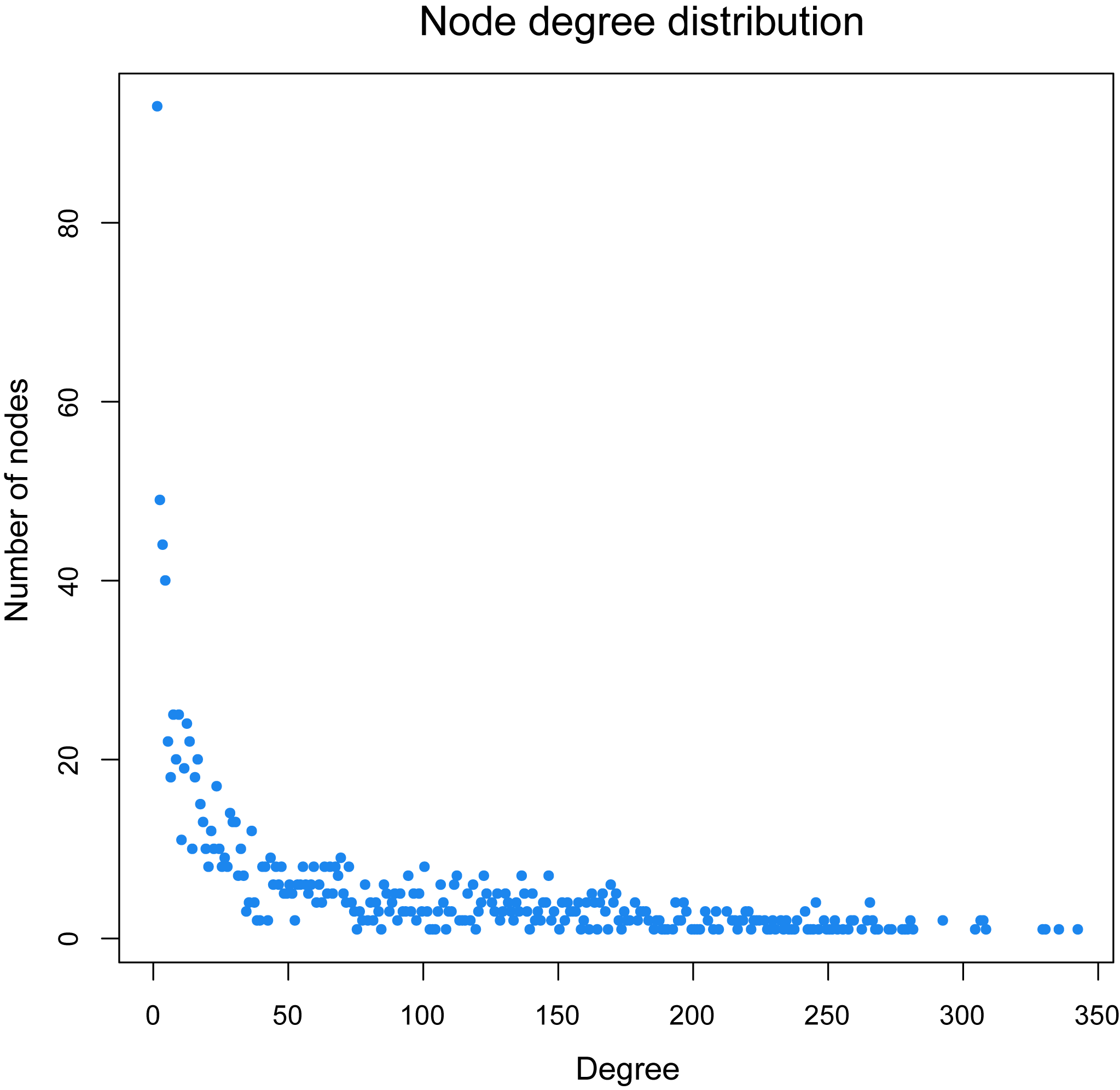

Supplement: Supplementary file 16 — Additional file 16: Figure S16. The node degree distribution of the DNA methylation correlation network. [file 12885_2019_6455_MOESM16_ESM.tif]

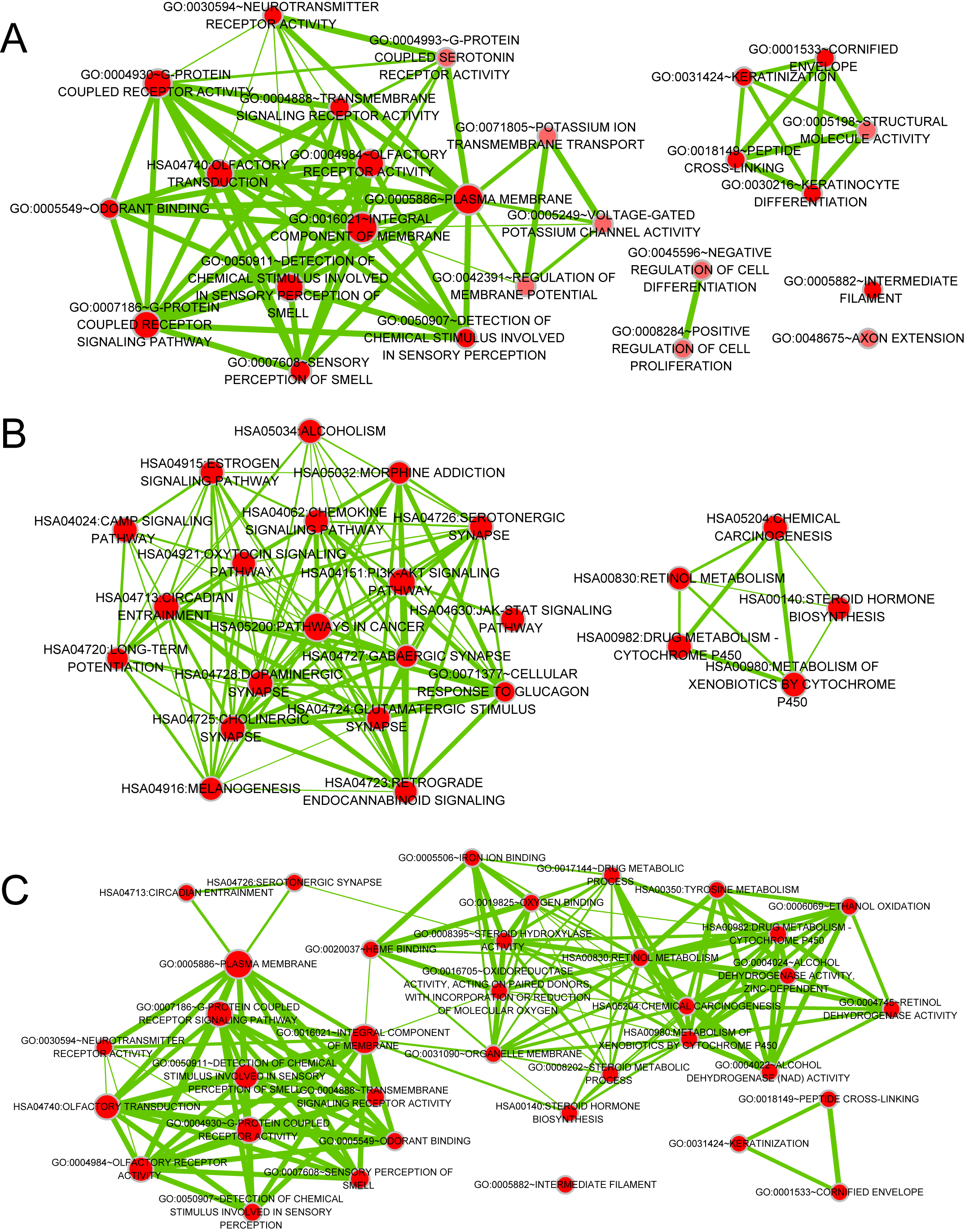

Supplement: Supplementary file 17 — Additional file 17: Figure S17. Enrichment analysis of key genes in DNA methylation network. A. Enrichment analysis of key genes in DNA methylation correlation network. B. Enrichment analysis of key genes in KEGG pathway network. [file 12885_2019_6455_MOESM17_ESM.tif]

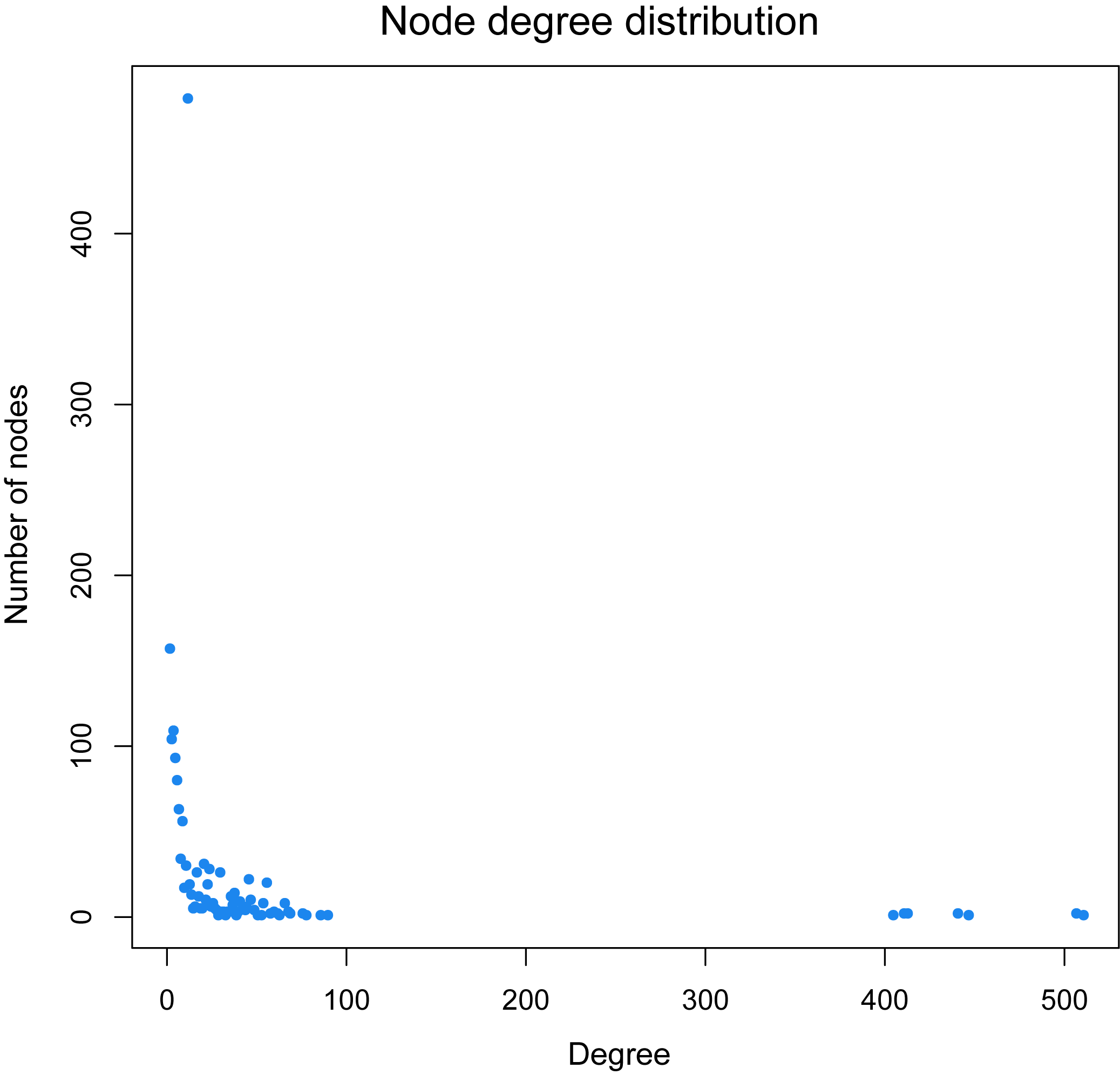

Supplement: Supplementary file 18 — Additional file 18: Figure S18. The node degree distribution of the KEGG pathway network. [file 12885_2019_6455_MOESM18_ESM.tif]

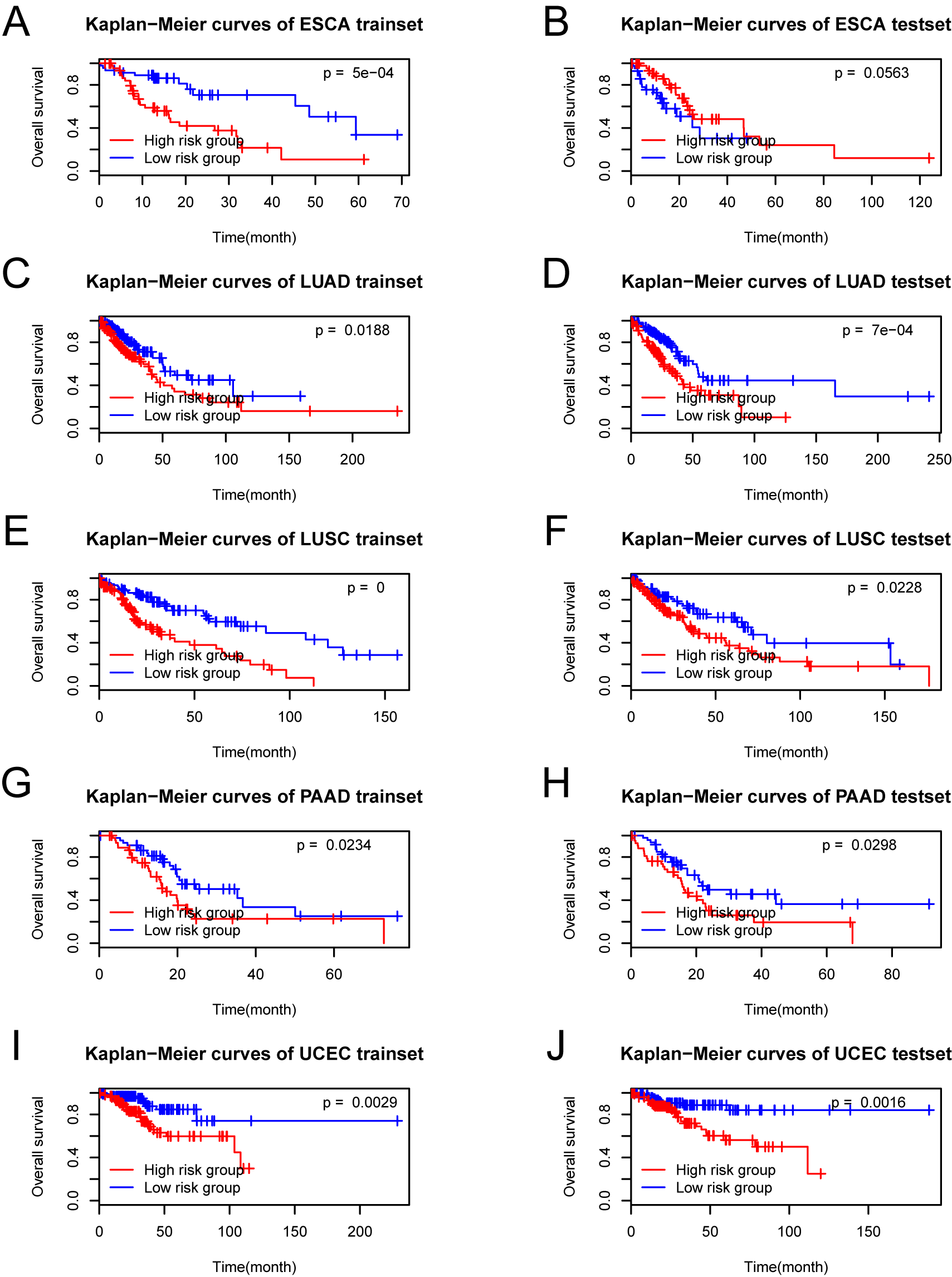

Supplement: Supplementary file 19 — Additional file 19: Figure S19. Kaplan-Meier survival curve. A. Survival curve of ESCA training set. B. Survival curve of ESCA test set. C. Survival curve of LUAD training set. D. Survival curve of LUAD test set. E. Survival curve of LUSC training set. F. Survival curve of LUSC test set. G. Survival curve of PAAD training set. H. Survival curve of PAAD test set. I. Survival curve of UCEC training set. J. Survival curve of UCEC test set. [file 12885_2019_6455_MOESM19_ESM.tif]
